# Expression Analysis of *Lrrk1*, *Lrrk2* and *Lrrk2* Splice Variants in Mice

**DOI:** 10.1371/journal.pone.0063778

**Published:** 2013-05-10

**Authors:** Florian Giesert, Andreas Hofmann, Alexander Bürger, Julia Zerle, Karina Kloos, Ulrich Hafen, Luise Ernst, Jingzhong Zhang, Daniela Maria Vogt-Weisenhorn, Wolfgang Wurst

**Affiliations:** 1 Helmholtz Center Munich, German Research Center for Environmental Health, Institute of Developmental Genetics, Neuherberg, Germany; 2 Technische Universität München, Center of Live and Food Science, Freising-Weihenstephan, Germany; 3 Max Planck Institute of Psychiatry, Munich, Germany; 4 German Center for Neurodegenerative Diseases, site Munich, Munich, Germany; University of Pittsburgh, United States of America

## Abstract

Missense mutations in the leucine-rich repeat kinase 2 gene (*LRRK2*) are linked to autosomal dominant forms of Parkinson’s disease (PD). In order to get insights into the physiological role of *Lrrk2*, we examined the distribution of *Lrrk2* mRNA and different splice variants in the developing murine embryo and the adult brain of *Mus musculus.* To analyse if the *Lrrk2*-paralog, *Lrrk1,* may have redundant functions in PD-development, we also compared *Lrrk1* and *Lrrk2* expression in the same tissues. Using radioactive *in situ* hybridization, we found ubiquitous expression of both genes at low level from embryonic stage E9.5 onward, which progressively increased up until birth. The developing central nervous system (CNS) displayed no prominent *Lrrk2* mRNA signals at these time-points. However, in the entire postnatal brain *Lrrk2* became detectable, showing strongest level in the striatum and the cortex of adult mice; Lrrk1 was only detectable in the mitral cell layer of the olfactory bulb. Thus, due to the non-overlapping expression patterns, a redundant function of *Lrrk2* and *Lrrk1* in the pathogenesis of PD seems to be unlikely. Quantification of *Lrrk2* mRNA and protein level in several brain regions by real-time PCR and Western blot verified the striatum and cortex as hotspots of postnatal *Lrrk2* expression. Strong expression of *Lrrk2* is mainly found in neurons, specifically in the dopamine receptor 1 (DRD1a) and 2 (DRD2)-positive subpopulations of the striatal medium spiny neurons. Finally, we identified 2 new splice-variants of *Lrrk2* in RNA-samples from various adult brain regions and organs: a variant with a skipped exon 5 and a truncated variant terminating in an alternative exon 42a. In order to identify the origin of these two splice variants, we also analysed primary neural cultures independently and found cell-specific expression patterns for these variants in microglia and astrocytes.

## Introduction

Parkinson’s Disease (PD), as the second most common neurodegenerative disorder, affects 1–2% of the population over the age of 65 (rising to 4–5% of the population from the age of 85) [1]–[3]. Even though the majority of PD cases are sporadic (>90%), several familial forms of the disease – which could be linked to a single genetic factor – were identified over the last fifteen years [4], [5]. Missense mutations in the *LRRK2* gene (dardarin), which are linked to changes in the coding region of the PARK8 locus [6]–[8], represent the most common cause of familial (up to 10% overall; up to 40% in certain ethnic populations) and sporadic (1–2%) late-onset forms of PD [7]–[14]. In contrast to hereditary loss-of-function PD-forms, *LRRK2* missense mutations do not interfere with the expression of this large multi-domain protein. Nevertheless, the activity of distinct protein domains is altered. The neuropathology of LRRK2-associated PD is heterogeneous and features the classical nigral degeneration with or without the presence of Lewy bodies and ubiquitin-positive or tau-positive inclusions [8], [15]–[18]. The *Lrrk2* gene is comprised of 51 exons coding for a large 2527 amino acid multi-domain protein [7]. It shares high homology with its slightly shorter paralog *Lrrk1*– another member of the ROCO protein kinase superfamily [19], [20]. Both proteins contain two domains, Roc (Ras in complex proteins, belonging to the Ras/GTPase family) and COR (C-terminal of Roc) characteristic of ROCO proteins [19], as well as the catalytic kinase domain with close sequence homology to MAPKKKs of the MLK (mixed lineage kinases)-type. In addition, several other domains are found in LRRK1 and/or LRRK2: LRRK2-specific repeats (only in LRRK2), ankyrin repeats (in both) and leucine-rich repeats (LRR, in both) plus a WD40 domain towards the C-terminus (only in LRRK2) [8], [20], [21]. Although mutations are spread over the whole *LRRK2* gene, most of the pathogenic mutations are found either in the kinase (G2019S, I2020T), COR (Y1699C) or Roc-GTPase domain (R1441C/G/H). *In vitro* data suggests that GTP binding promotes LRRK2 kinase activity and enhances neurotoxicity, indicating a gain-of-function mechanism in the etiology of PD [22]–[26]. A similar intramolecular, regulatory mechanism has also been reported for LRRK1 [27], but interestingly, no mutation clearly segregating with PD could be identified in this gene so far [28], [29]. Nevertheless, due to the highly conserved protein structures of both proteins it has been proposed that there might be a redundancy in their, up to now still unknown, physiological functions [30], [31]. Thus, detailed analysis of the organ and brain distribution of *Lrrk1* and *Lrrk2* could provide valuable hints towards their function. Therefore, in this study a series of detailed and comparative expression analyses for mammalian *Lrrk1* and *Lrrk2* was performed in mouse. Different analytical methods using samples from early stages of murine embryonic development until adulthood were used for this including: (i) Localization and expression level of *Lrrk1* and *Lrrk2* were analysed by radioactive *in situ* hybridization (ISH) in sections of whole embryos, postnatal, adult and aged brains. (ii) Expression level of *Lrrk2* mRNA were quantified by real-time PCR and protein level by Western blot in several brain regions. (iii) *Lrrk2* co-expression in different neuronal populations of the striatum and cortex were analysed by immunohistological stainings. (iv) In addition, two splice-variants of *Lrrk2* were identified in RNA samples from different adult brain regions and organs. Taken together, this is the first time that *Lrrk1* and *Lrrk2* expression (localization, expression level, cellular distribution and splicing) were analysed using a comparative approach with a number of independent highly sensitive analytical methods (qualitative and quantitative) in parallel. This combination of high-resolution ISH images; region-specific quantification of mRNA and protein level by real-time PCR and Western blot respectively; immunohistochemical analysis of the distribution in neuronal populations and the identification of cell-type specific splice variants is the basis for new insights into potential *Lrrk2* function.

## Materials and Methods

### Animals Housing and Tissue Preparation

All animal work was carried out in accordance with the European Communities’ Council Directive 2010/63/EU. All efforts were made to minimize animal suffering during the work. All protocols involving animal handling were approved by the committee for the Care and Use of Laboratory animals of the Government of Upper Bavaria, Germany. C57BL/6J mice (obtained from Charles River Germany) were group housed in individually ventilated type IIL cages (four mice per cage) and maintained on a 12 h/12 h light/dark cycle with food and water available *ad libitum*.

Mice were sacrificed and immediately thereafter intracardially perfused with 200 ml of ice-cold paraformaldehyde (PFA, 4%, pH 7.5). For post-fixation, the brains were incubated in PFA for 1–2 hours at RT. For embryo preparation, mothers were sacrificed via cervical dislocation. Embryos were dissected and immediately decapitated. Brains were dissected and immersion fixed in PFA overnight at 4°C. For embedding, brains were dehydrated through an ascending ethanol series (30%, 50%, 75%, 85%, 95% and 2×100%; 90 min per step), clarified for 2×60 min in 100% RotiHistol® (Carl Roth GmbH, #6640) at RT, equilibrated for 60 min in a 1∶1 mixture of RotiHistol®/paraffin at 65°C, transferred for 60 min into 100% paraffin at 65°C and incubated for 8 h in 100% paraffin at 65°C. At the end, samples were cooled down to room temperature in 100% paraffin (embedding) and stored at 4°C until cutting. Paraffin embedded brain tissue were cut on a Cryostat (MICROM, Heidelberg, Germany) in three different planes (coronal, sagittal and horizontal), put into a water bath (37–42°C) for flattening and mounted on SuperFrost® Plus slides (Menzel-Gläser, #J1800AMNZ), dried overnight on a heating plate and/or in an incubator at 37°C and stored at 4°C until use.

### Western Blot Analysis

LRRK2 expression analysis on protein level was performed using total protein samples from different brain as previously described by Gloeckner *et al*. [32] After blotting, bands were detected by monoclonal rat anti-Lrrk2 antibodies (1E11, produced by E. Kremmer, [32]–[36]) and HRP-coupled anti-rat secondary antibodies (Jackson Immunoresearch, #112-035-044). For measuring protein loading, mouse anti-β-actin antibodies (GeneTex, #GTX26276) and HRP-coupled anti-mouse secondary antibodies (Jackson Immunoresearch, #115-035-003) were used. Bands were visualized using the ECL detection kit (GE Healthcare).

### *In situ* Hybridization

ISH has been performed on paraffin sections using either [^35^S]-UTP-radiolabeled (Perkin Elmer) or digoxigenin (DIG) labeled riboprobes according to manufacturer’s protocol (Roche). The applied protocol is based on the published protocol of Dagerlind and colleagues [37]. In brief, 8 µm thick paraffin sections were rehydrated, partially digested with proteinase K, acetylated, dehydrated again and prehybridized in 100 µl hybridisation mix (containing 50% deionized formamide, 4× SSC, 0.5× Denhardt’s solution, 1% N-lauroylsarcosine, 20 mM Na-phosphate buffer and 10% Dextran sulphate) per slide for 1 h at 58°C. Following that, the slides were incubated with either [^35^S]-UTP-radiolabeled (1·10^6^ counts per million/slide) or DIG-labeled riboprobes (15–30 ng/slides) in 100 µl hybridisation mix per slide overnight at 58°C. Washing was performed in decreasing concentrations (4× to 0.1×) of saline sodium citrate (SSC) at RT, except the final washing step in 0.1× SSC for 2×30 minutes at 65°C). For the radioactive ISH, the hybridized slides were dehydrated and dipped in autoradiographic emulsion (Kodak NTB2), developed after 4–6 weeks and counterstained with cresyl violet according to standard protocols. For detecting DIG-labeled riboprobes, an anti-DIG antibody coupled to the alkaline phosphatase (AP) enzyme has been used; subsequent either nitro blue tetrazolium and 5-bromo-4-chloro-3-indolyl phosphate (Boehringer Mannheim, Germany) or a tyramide–cyanine 3 TSA kit (Perkin Elmer Life Sciences, Boston, USA) have been used as chromophore. Riboprobes were obtained from cDNA fragments cloned into the pCR™II-TOPO® vector (Life Technologies, Carlsbad, USA) by *in vitro* transcription by RNA polymerase T3, T7 or SP6, either utilizing [^35^S]-UTP (Perkin Elmer Life Sciences, Boston, USA) or DIG-labeling mix according to manufacturer’s protocol (Roche, Mannheim, Germany). To obtain specific ISH-probes for *Lrrk1* and *Lrrk2*, several overlapping and non-overlapping cDNA fragments have been amplified from murine total mRNA by RT-PCRs. Sequences of used primers can be found in (Figure S9).

### Immunhistochemistry on Paraffin Sections

IHC on paraffin sections was performed in coplin jars except for blocking reactions, antibody incubations, incubation with ABC-reagent and DAB-stainings. Glass slides were dewaxed for 45 min in xylol, rehydrated in a series of descending alcohol (100%, 96% and 70%) and rinsed with water. To destroy endogenous peroxidases, slides were incubated for 5 min in 0.1% H_2_O_2_ (in PBS) before blocking for 1 h in 10% FCS (with 0.05% Triton-X in PBS). Primary antibodies against DARPP-32 (Abcam, ab40801), dopamine D1 receptor (Calbiochem, #324390), dopamine D2 receptor (Chemicon, AB5084P), NeuN (Chemicon, MAB377) were diluted in 10% FCS (with 0.05% Triton-X in PBS) and incubated overnight at 4°C in a humid chamber. Appropriate secondary antibodies were then applied on the next day diluted in 10% FCS (with 0.05% Triton-X in PBS) for 1 h at RT in a humid chamber. Next, the DAB working solution (Sigma) was freshly prepared (1 ml DAB stock solution, 15 µl 30% H_2_O_2_ and 19 ml 0.1 M Tris-HCl, pH 7.4) and applied to the sections for 30 min in a humid chamber. Finally, sections were dehydrated in a series of descending alcohol (70%, 96% and 100%) and xylol and embedded in Roti-HistoKit II (Carl Roth GmbH).

### Reverse Transcriptase and Quantitative Real-time PCR

RT-PCR was performed on Trizol (Invitrogen, #15596-026) or RNeasy Mini Kit (Quiagen) extracted RNA samples from C57CL/6J mice. Reverse transcription was performed using random hexamers and SuperScript®-II (Invitrogen, #12371-019) or SuperScript®-VILO (Invitrogen, #11904-018) according to the manufacturer’s instructions.

Quantitative Lrrk2 expression analysis was performed using total RNA from different brain regions and the ABI PRISM 7900 Sequence Detection System (Applied Biosystems) and TaqMan® reaction mixes for endogenous *Lrrk2* (Mm00481934_m1, Applied Biosystems) and β-actin (NM_007393.1, Applied Biosystems). All samples were measured in triplicates using the following PCR program: an initial step of 95°C for 10 min and then 40 cycles of 95°C for 15 s and 60°C for 1 min. For quantification of Lrrk2 transcripts, three different qPCR reactions were performed from each cDNA sample (endogenous LRRK2, alternative LRRK2 and β-actin control). A 20 µl reaction contained 10 µl of TaqMan® Universal PCR Master Mix (Applied Biosystems), 2 µl cDNA template, 300 nmol/L of each primer and 200 nmol/L of the specific probe. Sequences of used primers and probes can be found in (Figure S9). For the β-actin control reaction an ACTB TaqMan® Gene Expression Assay Mix Mouse (Applied Biosystems) was used. No template controls (NTC) were included in each assay and amplification and detection were performed under standard conditions as recommend by Applied Biosystems. The fluorescent signal intensities were recorded and analysed during PCR amplification using the Sequence Detection Software (SDS) software (Applied Biosystems). To determine the LRRK2 ratios, all values were initially normalized to the β-actin expression. Following, the ratio of variant versus endogenous *Lrrk2* was determined (2^−[ΔΔCTvariant−ΔΔCTnormal]^).

### Preparation of Primary Neurons, Microglia and Astrocytes

Primary cortical neuronal cultures of C57BL/6J brains were generated as previously described [38]. For preparation of primary astrocyte and microglia cultures, newborn pups (1–3 days old) were decapitated, brains were dissected free of the meninges, and the cerebral cortices were isolated. Then, cortices were mechanically dissociated and cells were resuspended in astrocyte [DMEM (Invitrogen) supplemented with 10% FCS, 1% Pen/Strep, 10 ng/ml human EGF (Invitrogen) and murine bFGF (Peprotech)] or microglia medium [DMEM/F12 (Invitrogen) supplemented with 10% FCS and 1% Pen/Strep], respectively. Culture was performed in 6-well dishes at 5% CO_2_ and medium was changed every 3–4 days. To deplete as many microglia as possible, plates were shaken rapidly (15–30 s) prior to each medium change (step done only for astrocyte cultures). After confluency was reached, cultures were trypsinated using a 1∶3 dilution of 0.25% trypsin and serum-free medium until the astrocyte containing cell layer slowly detached (while the microglia stayed attached to the plate) [39]. To harvest astrocytes, the floating cell-layer were transferred to a new plate and dissociated by gentle pippeting for further subculture. To harvest microglia, just new medium was added to the old plates after astrocyte-removal and cells were used after 3–6 days. The astrocyte-subculture procedure was repeated 3 times until cultures were taken for experiments and mitogens were removed from the medium 4 days before RNA was isolated.

## Results

### Comparative Expression Analysis of *Lrrk1* and *Lrrk2* during Murine Embryonic Development

The pattern of expression reflects the physiological role of a protein. Thus, a comparative expression analysis of *Lrrk2* and *Lrrk1* was performed in C57BL/6J mice by radioactive ISH. This was also to evaluate the possibility of redundant functions between these two highly conserved paralogs. The main emphasis of this expression analysis was put on the brain of adult mice; embryonic development was also taken into account.

**Figure 1:**
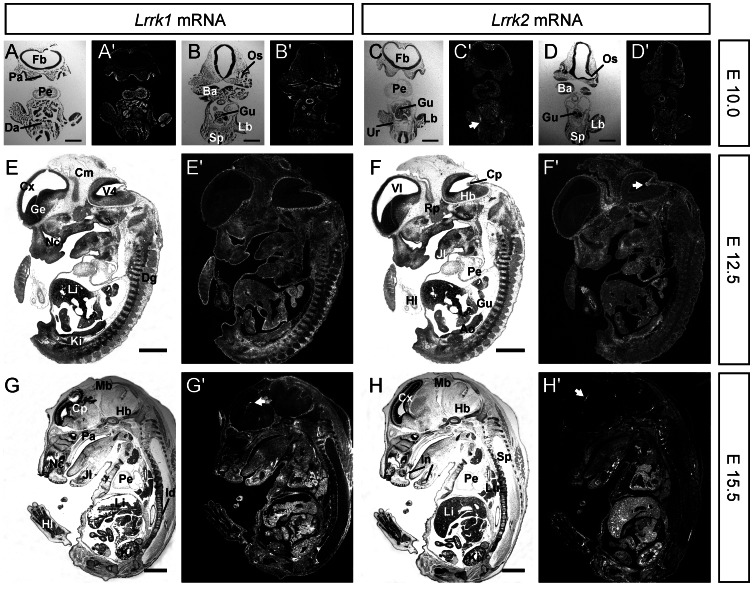
Comparative expression analysis of *Lrrk1* and *Lrrk2* mRNA during embryogenesis. ISH for *Lrrk1* (left part) and *Lrrk2* (right part) mRNA in sections from E10, E12.5 and E15.5 embryos. For each embryo, a brightfield image (e.g. A, for anatomical orientation) and a darkfield image (e.g. A’, ISH signals in white) are shown. (**A–D**) At E10, small hotspots of *Lrrk1* expression were observed only in the cephalic mesenchyme (Cm in A+B), the palate and the optic stalk (Oc in B), while *Lrrk2* mRNA was found basically in the urogenital ridge (Ur in C, arrow). Note that at this stage of gestation expression of *Lrrk1* and *Lrrk2* was virtually absent from the developing CNS (A–D). (**E,F**) At E12.5, additional spots for *Lrrk1* expression were visible in the epithelia of the nose and mouth (Nc in E) and for *Lrrk2* expression in the choroid plexus (Cp in F, arrow) and developing pituitary gland (Pi in F). (**G,H**) Around E15.5, *Lrrk1* and *Lrrk2* expression became stronger in several organs of the embryos (G+H) including liver (Li), kidney (Ki), lung (Lu) and heart (Pe) as well as in the choroid plexus (Cp, arrows). Ao, Aorta; Ba, branchial arch; Cm, cephalic mesenchyme; Cx, cortex; Cp, choroid plexus; Da, dorsal aorta; Dg, dorsal root ganglia; Fb, forebrain; Ge, ganglionic eminence; Gu, gut; Hb, hindbrain; Hl, hind limb; Id, intervertebral disc; In, incisive; Jl, lower jaw; Ki, kidney; Lb, limb but; Li, liver; Lu, lung; Mb, midbrain; Nc, nasal cavity; Oc, optic cup; Os, optic stalk; Pa, palate; Pe, pericardium; Rp, Rathke’s pouch; Sp, spinal cord; Ur, urogenital ridge; V4, fourth ventricle; Vl, lateral ventricle. Orientation of sections: A+C, coronal; B, horizontal; D–H, sagittal. Scale bars represent 500 µm in A–D, 1 mm in E–H.

*Lrrk2* mRNA was first analysed using semi-quantitative RT-PCR in undifferentiated embryonic stem (ES) cells and in early stages of embryonic development (E8.5 to E12.5 embryos) (Figure S1A). In virtually all developmental stages and organs (from ES cells until adulthood), expression of *Lrrk2* mRNA could be detected (Figure S1A and data not shown). The earliest embryonic stage, which was analysed by ISH, was E7.5 (Figure S1B). At this developmental stage only the surrounding maternal tissue (i.e. the parietal yolk sac) exhibited strong *Lrrk2* expression, whereas signals in the ectodermal, mesodermal and endodermal embryonic tissues were too weak to be detected even by radioactive ISH (Figure S1B). At embryonic stages E9.0 to E11.5, weak expression of *Lrrk1* and *Lrrk2* was observed widespread over the entire embryo beside the neuroepithelium of the developing CNS. At stage E10, *Lrrk1* showed distinct areas of stronger expression in the cephalic mesenchyme tissue and palate (Figure 1A,B), the inner layer of the optic cup (the future nervous layer of the retina), in the tissue of the first brachial arch and in the developing gut (Figure 1B), ). *Lrrk2* was expressed at higher level besides the head mesenchyme mainly in the urogenital ridge (Figure 1C,D). At stage E12.5, further *Lrrk1* expressing domains became visible in the epithelia of the nose and mouth (Figure 1E) and significant *Lrrk2* expression was observed in Rathke’s pouch as part of the developing pituitary gland (Figure 1F). Again, *Lrrk1* and *Lrrk2* could not be detected in the developing central nervous system, however, one hot spot of expression was the choroid plexus. Furthermore it is noteworthy that both genes were strongly expressed in the meninges – a non-neuronal tissue directly surrounding the developing central nervous system (CNS) (Figure 1E,F). At stage E14.5 (Figure S1C–E for *Lrrk2*) and E15.5 (Figure 1G,H), robust *Lrrk1* and *Lrrk2* expression was detected in parts of the developing kidney, lung, liver and heart (Figure 1G,H). Interestingly, the expression of *Lrrk1* and *Lrrk2* at this developmental stage showed a high degree of overlap, however, the localization within the tissues was diverse in some cases. For example, *Lrrk2* mRNA in the developing kidney was more or less restricted to structures like the metanephric vesicles (Figure S1E), which was not the case for *Lrrk1* expression (data not shown). In agreement, both *Lrrk1* and *Lrrk2* were still strongly expressed in the choroid plexus (Figure 1G,H and Figure S1C), while no expression was observed in other parts of the embryonic brain until birth (data not shown). Taken together, the expression pattern of *Lrrk1* and *Lrrk2* were largely overlapping during embryogenesis in many organs and structures. However, *Lrrk1* expression seemed to be stronger and more widespread than expression of *Lrrk2*.

### Comparative Expression Analysis of *Lrrk1* and *Lrrk2* in the Murine Postnatal, Adult and Aged Brain

**Figure 2:**
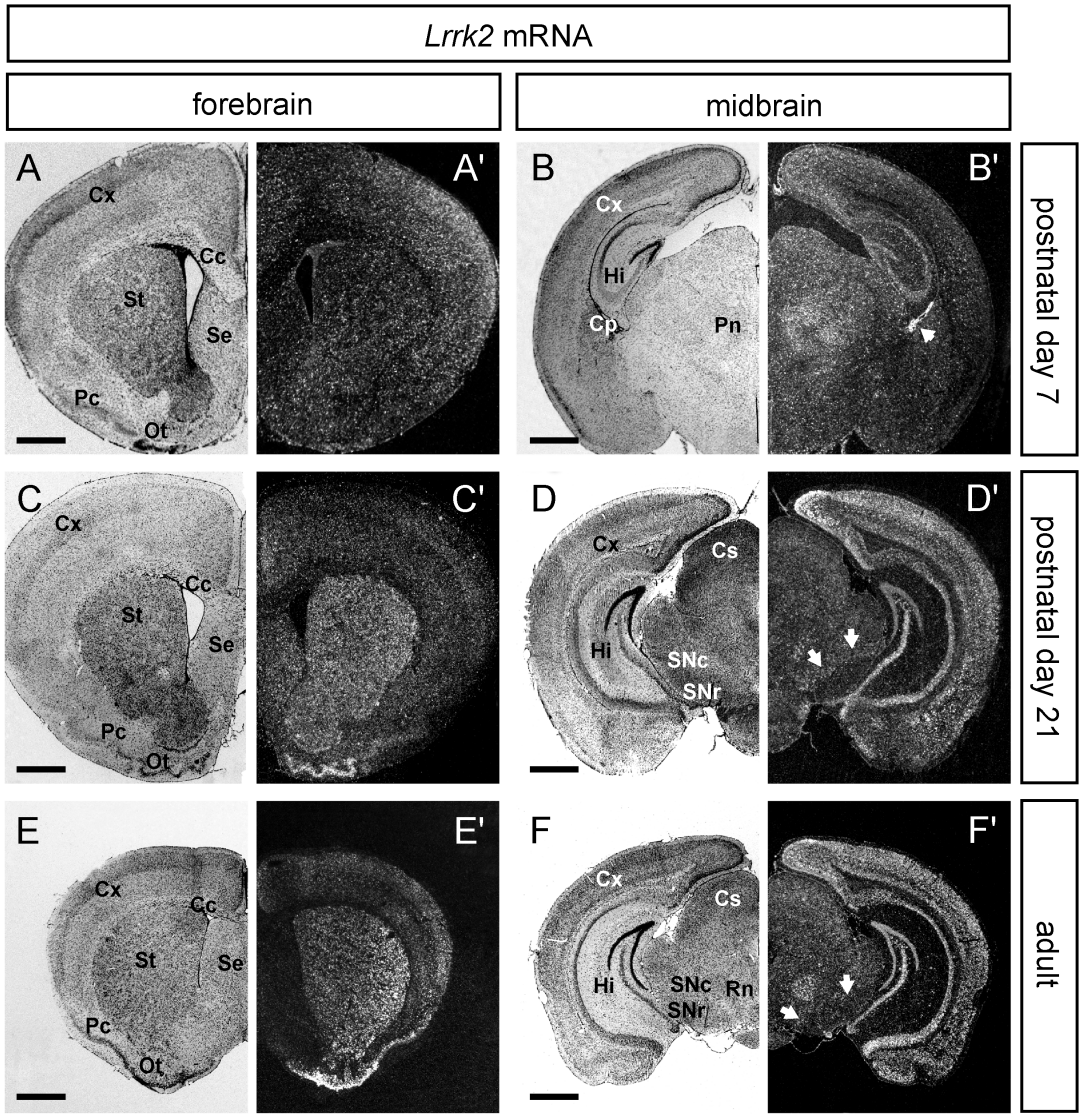
Expression analysis of *Lrrk2* mRNA in the forebrain and midbrain of postnatal mice. ISH for *Lrrk2* mRNA in coronal sections of forebrain (left part) and midbrain (right part) from postnatal day 7, postnatal day 21 and adult mice. Note that expression of *Lrrk2* is highly dynamic in the postnatal forebrain. While the expression level in the striatum and the olfactory tubercle seem to increase dramatically during development, the *Lrrk2* level in cortex remain rather unchanged (**A,C,E**). On the level of the midbrain, ISH signals for *Lrrk2* augment considerably in the hippocampus and cortex, while the level in midbrain structures like the Substantia nigra pars compacta (white arrows, SNc) remain quite low (**B,D,F**). Abbreviations: Cc, corpus callosum; Cx, cortex; Cp, choroid plexus (white arrowhead in B’); Cs, superior colliculus; Hi, hippocampus; Ot, olfactory tubercle; Pc, piriform cortex; Pn, parafascicular nucleus; Rn, red nucleus; Se, septum; SNc, SN pars compacta; SNr, SN pars reticulata; St, striatum. Scale bars represent 1 mm.

**Figure 3:**
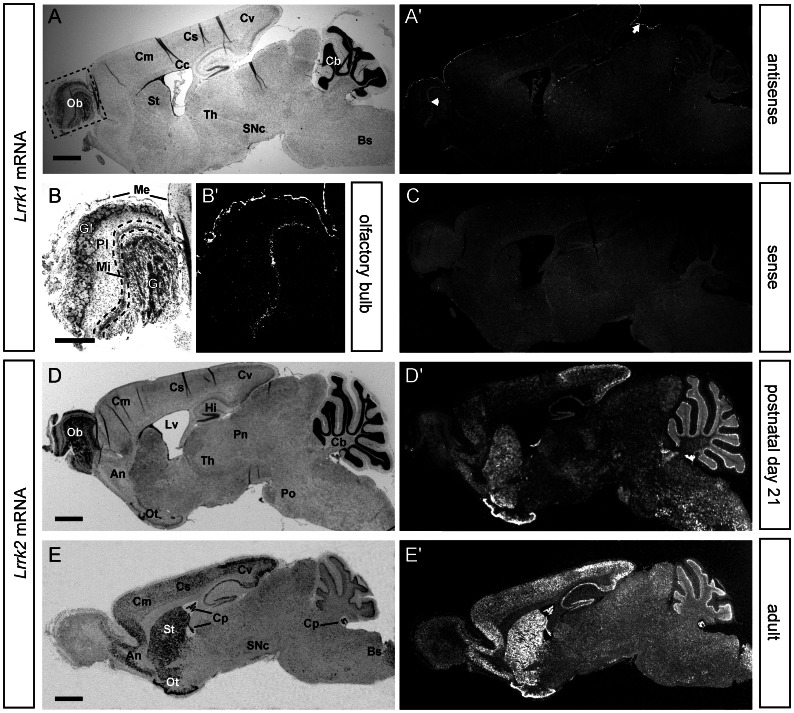
Comparative expression analysis of *Lrrk1* and *Lrrk2* mRNA in the brain of adult mice. ISH for *Lrrk1* (top part) and *Lrrk2* (bottom part) mRNA in sections from P21 (**A–D**) and adult mice (**E**). Note that *Lrrk1* mRNA is barely detectable in the adult mouse brain and only visible in the non-neuronal meninges (white arrow) and the olfactory bulb (white arrowhead) (**A**). A detailed view onto the adult olfactory bulb depicts the solely neuronal expression of *Lrrk1* in the mitral cell layer (**B**). Specificity of the *Lrrk1* signals were verified by using the corresponding sence-probe as negative control (**C**). In contrast, strong *Lrrk2* expression can be detected in various regions throughout the postnatal (**D**) and adult CNS (**E**). Abbreviations: An, anterior olfactory nucleus; Bs, brain stem; Cb, cerebellum; Cc, corpus callosum; Cm, motor cortex; Co, cortex; Cp, choroid plexus; Cs, somatosensory cortex; Cv, visual cortex; Gl, glomerular layer; Gr, granual layer; Hi, hippocampus; Me, meninges; Mi, mitral layer; Ob, olfactory bulb; Ot, olfactory tubercle; Pn, parafascicular nucleus; Pl, plexiform layer; Po, pons; SNc, substantia nigra pars compacta; St, striatum; Th, thalamus; Lv, lateral ventricle. Scale bars represent 1 mm (A, D, E) and 500 µm (B).

Distinct expression of *Lrrk2* in neural cells of the CNS were observed around birth (Postnatal day 0, P0), where weak *Lrrk2* expression was found in the entire brain, specifically in the developing cortex, cerebellum and brain stem (Figure S2A). During postnatal development until P7, the overall neuronal ISH signals but also the expression in distinct domains like cortex, striatum and olfactory bulb increased notably (Figure S2B). Between P7 and P21, the level of *Lrrk2* expression in the forebrain and midbrain varied in a highly dynamic manner. As an example, *Lrrk2* ISH signals in the striatum increased in the forebrain and surpassing the cortical expression level, which remained rather unchanged (Figure 2A,C,E). Also, the level of *Lrrk2* increased considerably in hippocampus and posterior cortex, while expression in the substantia nigra (SN) was constantly low (Figure 2B,D,F). In contrast to *Lrrk2* (Figure 3D), *Lrrk1* mRNA was barely detectable in the postnatal brain, namely at P21. At this time-point of development, only the non-neuronal meninges and the mitral cell layer of the olfactory bulb showed faint ISH signal for the *Lrrk1* specific probe (Figure 3A and Figure S4I).

In the adult (older than 6 weeks) mouse brain, strong *Lrrk2* ISH signals were present all over the brain, with highest level in the forebrain, the olfactory tubercle and the striatum (Figure 3E and Figure S4B). In addition, strong *Lrrk2* expression was also observed in the motor, somatosensory and visual cortex (Figure S3), as well as in the anterior olfactory nucleus and the hippocampus. Herein, specifically the pyramidal cells of the hippocampus proper (CA1, CA2 and CA3) as well as the dentate gyrus were robustly labeled (Figure 3E and Figure S4F). *Lrrk2* expression was also found in the subventricular zone (Figure S4D) – the second region of adult neurogenesis besides hippocampus. Interestingly, the dopaminergic system of the ventral midbrain (built up by the SN pars compacta and pars reticulata, as well as the ventral tegmental area) showed only comparable low level of *Lrrk2* expression (white arrows in Figure 2D,F; Figure 3E and Figure S4H). In the hindbrain, strongest ISH signals were found in the purkinje cell layer and molecular layer of the cerebellum (Figure 3E and Figure S4J). In the adult pituitary gland, *Lrrk2* can be observed in the anterior and especially in the intermediate lobe (data not shown) which constitute the adenohypophysis, but not in the posterior lobe (neurohypophysis). During ageing, no significant differences – neither in expression pattern nor in the expression level – were observed in up to 24 month old mice (data not shown). *Lrrk1* retained its postnatal expression pattern also in adulthood (Figure 3 and Figure S3–4).

Taken together, the expression of *Lrrk2* in the murine CNS became detectable around birth and increased during early postnatal development. In adult mice, the level of *Lrrk2* mRNA in the forebrain and hindbrain were higher than in the midbrain. The general *Lrrk2* expression patterns observed in six week old brains sustained stable during adulthood. In contrast to *Lrrk2*, *Lrrk1* was barely detectable in the adult mouse brain and only present in the mitral layer cell layer in the olfactory bulb where no prominent *Lrrk2* expression can be found.

### Quantitative Analysis of *Lrrk2* mRNA and Protein Level in the Adult CNS

**Figure 4:**
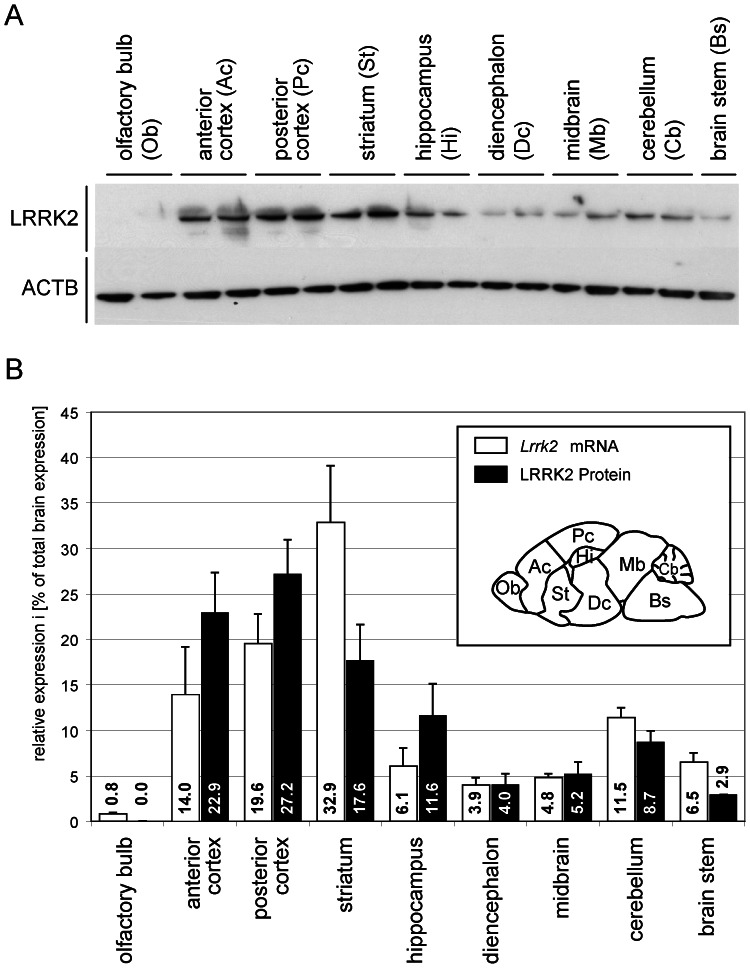
Expression analysis of *Lrrk2* mRNA and protein in various brain regions from adult mice. Quantifications of *Lrrk2* level in various brain regions by qPCR (mRNA) and Western blot (protein). Before calculation, LRRK2 protein level from Western blot analyses (**A,** representative Western blot) were normalized to beta-actin (ACTB) as loading control and *Lrrk2* mRNA level from qPCR to a beta-actin control accordingly. (**B**) To allow a direct comparison between *Lrrk2* mRNA and protein level, the sum of all individual relative expression level were set to 100% in both cases ({percentage brain region}z. = {relative level brain region}/{sum of all relative level}×100%). The definition of brain regions used for this analysis is indicated in the figure legend (B). Abbreviations: Ac, anterior cortex; Bs, brain stem; Cb, cerebellum; Dc, diencephalon; Hi, hippocampus; Mb, midbrain; Ob, olfactory bulb; Pc, posterior cortex; St, striatum.

Since *Lrrk1* was barely detectable in the adult mouse brain, all further analyses were focused on *Lrrk2*. To obtain quantitative data with regard to LRRK2 expression level in the adult brain, we performed quantitative PCR (qPCR) and Western blot analyses on total RNA and protein samples from different brain regions (Figure 4A). Therefore, mouse brains were separated into their two hemispheres: one half was used for Western blot and the other for qPCR analysis. These were separated into nine different brain regions and utilized to prepare total RNA or protein samples. To allow a direct comparison between *Lrrk2* mRNA and protein level, the relative expression level for all brain regions have been summed up and set to 100%. Interestingly, the pattern of protein expression found by Western blot matched the mRNA level observed by ISH (compare Figure 3 and Figure 4B). The highest LRRK2 protein level was found in samples from the posterior cortex (27.2% (±3.7%) of total LRRK2 protein in the adult brain), followed by striatum, hippocampus and cerebellum (Figure 4B). Also by qPCR, the highest *Lrrk2* mRNA level were found in samples from cortex and striatum, followed by cerebellum, brain stem and hippocampus (Figure 4B). Apart from striatum, there were no remarkable discrepancies between the relative protein and mRNA level in the different brain regions. However, the striatal mRNA level was 32.9% (±6.2%) of the total *Lrrk2* mRNA, while the very same region contained only 17.6% (±4.0%) of the total LRRK2 protein. A possible explanation for this phenomenon could be posttranscriptional regulation of LRRK2 in the striatum via specific microRNAs and/or transport of the protein. However, further analyses would be necessary to proof this hypothesis. Taken together, qPCR and quantitative Western blot analyses further verified the ISH-based expression pattern of *Lrrk2*.

### Analysis of *Lrrk2*-expressing Neuronal Subtypes

**Figure 5:**
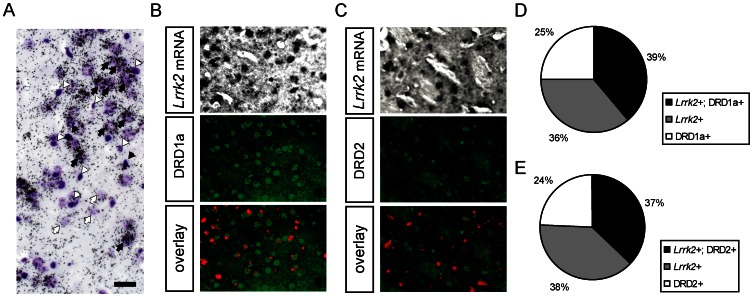
Cellular expression analysis of *Lrrk2* mRNA in the striatum of adult mice. (**A**) High magnification of a representative brightfield ISH image using radioactive-labeled *Lrrk2*-specific riboprobes and counterstained by cresyl violet: *Lrrk2* mRNA is predominantly expressed in neurons (black arrows, *Lrrk2*-positive neurons; white arrows, *Lrrk2*-negative neurons; black arrowheads, *Lrrk2*-positive glia; white arrowheads, *Lrrk2*-negative glia). (**B, C**) Representative images of double *in situ*−/Immunohistochemistry (ISH/IHC) in the medial part of the putamen: ISH for *Lrrk2* using DIG-labeled riboprobes (non-fluorescent black precipitate) followed by IHC stainings (green) for the two main dopamine receptors D1 (DRD1a) and D2 (DRD2). (**D, E**) Quantification of the *Lrrk2*/DRD1a and *Lrrk2*/DRD2 ISH/IHC stainings in the striatum revealed 36% *Lrrk2*-positive, 25% DRD1a-positive and 39% double positive cells. In case of the DRD2 *in situ*−/Immunohistochemistry, 38% *Lrrk2*-positive, 24% DRD2-positive and 37% double positive cells could be detected. Scale bar represents 25 µm.

ISH, Western blot and real-time PCR analyses revealed highest *Lrrk2* expression level in samples from cortex and striatum, wherein the last one is known to be the major target area of the dopaminergic neurons from the SN. Based on this observation we studied in detail the cellular expression of *Lrrk2* in the striatum by double in situ-hybridization/Immunohistochemical (ISH/IHC) stainings: on paraffin sections of the adult striatum nonradioactive ISH (nonfluorescent dark precipitate as signal indicating strong *Lrrk2* expression) were performed, followed by fluorescent IHC for different neuronal markers. High magnifications of radioactive ISH brightfield pictures gave further insights into the cellular expression of *Lrrk2*. High expression was associated with neurons as identified by their typical morphology in the cresyl violet (CV) counterstaining (black arrows in Figure 5A). Most but not all non-neuronal cells, marked by their more condensed appearance in CV stainings, were devoid of *Lrrk2* mRNA (white arrowheads in Figure 5A) We could confirm this finding by a high overlap of *Lrrk2* mRNA expressing cells with the neuronal marker NeuN (neuronal nuclei antigen) in striatal sections by ISH/IHC stainings (data not shown), indicating a predominant but not exclusive neuronal expression of *Lrrk2*. In addition, virtually all striatal cells strongly expressing *Lrrk2* mRNA were also positive for the dopamine and cAMP regulated phosphoprotein 32 (DARPP-32) (data not shown); a marker for medium spiny neurons (MSNs) in the striatum [40], suggesting that *Lrrk2* plays a functional role in dopaminoceptive neurons. Hence, we studied the cellular expression of *Lrrk2* mRNA in the two canonical dopaminergic circuits of the basal ganglia, the direct and indirect pathway, which are marked by the expression of either dopamine receptors D1 (DRD1a) or D2 (DRD2), respectively. The quantification of ISH/IHC for *Lrrk2* and DRD1a depicted in the striatum 36% of the cells being *Lrrk2*-positive, 25% DRD1a-positive and 39% double positive (Figure 5B,D). In case of the DRD2 ISH/IHC, 38% of the cells were solely *Lrrk2*-positive, 24% DRD2-positive and 37% double positive cells could be detected (Figure 5C,E). This data indicates that neither the direct nor the indirect pathway preferentially expresses *Lrrk2*. In addition it could be observed that only subpopulations show strong *Lrrk2* expression in both pathways.

### Analysis of *Lrrk2* mRNA Transcripts and Splice Variants

**Figure 6:**
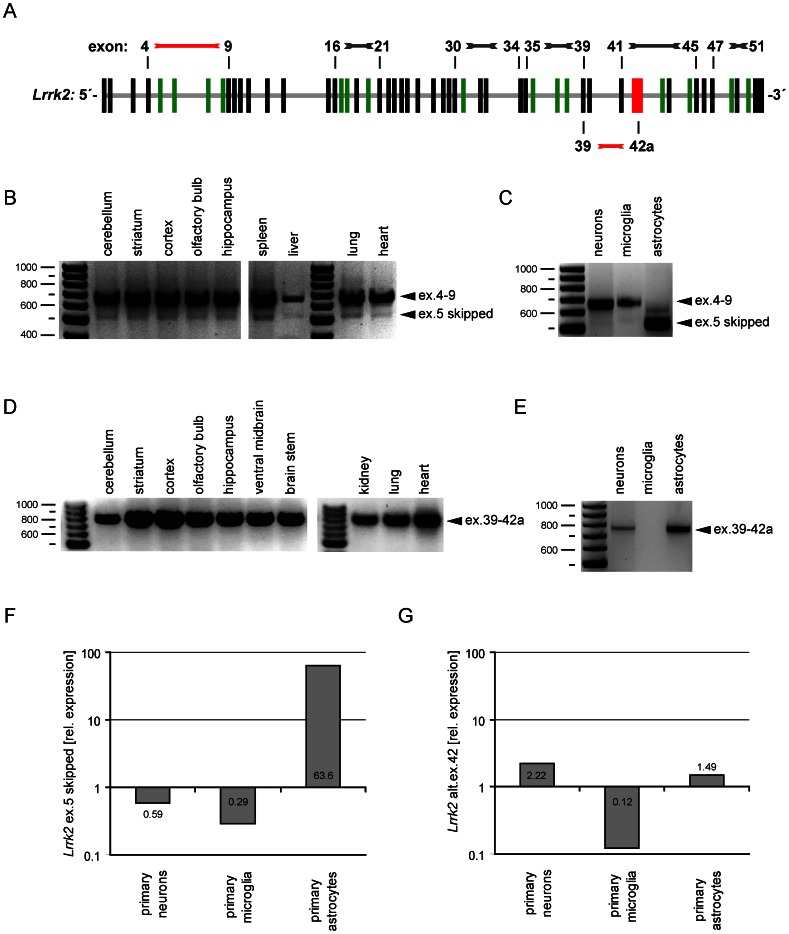
Qualitative expression analysis of alternative *Lrrk2* mRNA transcripts. RNA samples from different brain regions and organs were analysed by RT-PCR using a primer combination that amplifies *Lrrk2* transcripts between exon 4 and exon 9 (**A**). Note that there is – beside the band of the endogenous *Lrrk2* (ex.4–9) – an additional lower band visible in all samples analysed representing a mRNA where exon 5 is spliced out (ex.5 skipped) (**B**). The same primers were used to analyse RNA samples from primary neuronal cultures (i.e. neurons, microglia and astrocytes) (**C**). Interestingly, the band representing the splice variant without exon 5 (E5 skipped) was dominantly expressed in samples from astrocytes, while the band for the endogenous transcript (E4–E9) was almost completely missing in these cells. (**D**) RNA samples were analysed by RT-PCR using primers that amplify *Lrrk2* transcripts between exon 39 (E39) and an alternative exon 42a (ex.42a). Note that the alternative exon 42a is present in all samples analysed (ex.39–42a). (**E**) The same primers were used to analyse RNA samples from primary neuronal cultures (i.e. neurons, microglia and astrocytes). Interestingly, the alternative exon 42a could not be amplified from primary microglia cDNA. (**F**) Relative expression level as measured by quantitative RT-PCR with primers and probes specific for either the endogenous *Lrrk2* transcript (full-length *Lrrk2*), or for the alternative *Lrrk2* mRNA transcripts with skipped exon 5 (ex.5 skipped). The relative expression is depicted as a ratio between endogenous *Lrrk2* transcript and the alternative product on a logarithmic scale. (**G**) Accordingly for the alternative *Lrrk2* mRNA transcript ending in the alternative exon 42a (alt.ex.42), the relative expression level have been determined by quantitative RT-PCR with specific primers and probes for the alternative product and normalized to the expression of the endogenous *Lrrk2* transcript (full-length *Lrrk2*).

Alternative splicing increases the diversity of mRNAs expressed from the genome, which has profound functional effects [41]. Therefore, we completed the detailed *Lrrk1* and *Lrrk2* expression analysis by determining *Lrrk2* splice variants and their expression in various brain regions and organs from adult mice. Therefore, we first studied the genomic organization of *Lrrk2* for the presence of exons which could be spliced out without causing dramatic rearrangements of the residual *Lrrk2*-transcript (i.e. exons with a number of base-pairs that is divisible by three and thus can be skipped without causing a frame-shift in the residual mRNA coding sequence). Thereafter, we performed six different RT-PCR analyses using primer pairs amplifying the regions of exons 4–9, 16–21, 30–34, 35–39, 41–45 and 47–51 (Figure S5A). All reactions produced single amplicons representing the canonical *Lrrk2* mRNA (Figure S5B–F) except the primer combination for exons 4 to 9, which revealed an additional, smaller band of unknown origin (Figure 6B). We analysed RNA samples from the brain regions with the highest *Lrrk2* expression (striatum and cortex), but also cerebellum, olfactory bulb and hippocampus. In addition, samples from several organs were analysed using the same primers. Interestingly, all samples analysed showed this additional lower band in a comparable ratio. Sequence analysis of the fragments revealed a novel splice variant in which exon 5 (135bp) is absent (Figure S6). To test for cell-type-specific expression within the CNS, the same primers were used to analyse RNA samples from primary neuronal, microglia and astrocyte cultures, which all were prepared from cortical tissues of C57Bl/6J mice. As already suggested by the ISH expression analysis, the endogenous *Lrrk2* transcript could be detected in all three cell types with several primer pairs (data not shown). Using primers for exons 4–9, we found the same pattern in samples from neurons and microglia as observed in organs and brain tissues (Figure 6C). However, in astrocytes the splice variant without exon 5 was dominantly expressed whereas the full length transcript was barely detectable (Figure 6C). In addition, we performed a qPCR expression analysis using a probe located within exon 5 specific for the full-length *Lrrk2* transcript and a probe located in the junction between exon 4 and 6 specific for the alternative *Lrrk2* mRNA transcript with skipped exon 5 (Figure 6F). Putative differences in the sensitivity of the primer combinations and probes may not allow a direct quantitative comparison between endogenous and alternative form. Nevertheless, the results confirmed a pronounced expression of alternative *Lrrk2* mRNA transcript in primary astrocytes compared to primary neurons and microglia.

Next, we analysed all samples for the presence of an alternative exon 42a (containing several stop codons), which was already annotated in ensembl.org as part of an alternative processed transcript (ensembl.org sequence ENSMUST00000140734, Figure S7). In contrast to the full length *Lrrk2* transcript, which consists of 51 exons, 8275 base-pairs and 2527 amino acids (ensembl.org sequence ENSMUST00000060642), the annotated sequence encompasses only 19 of these exons (3452 base-pairs), starts in exon 24 and ends with the alternative exon 42a (Figure S5). There is no information available about the sequence which is 5′ of the annotated 19 exons. Thus, it could be possible, that this annotation is part of an alternative *Lrrk2* transcript, that starts regularly with exon 1, but ends with exon 42 (6456bp and 2152 amino acids long). To confirm the expression of this transcript, we performed RT-PCR analyses using primers amplifying the sequence between exon 39 and the alternative exon 42a (Figure 6D). Interestingly, expression of this alternative exon 42a could be identified in all brain regions and organs analysed. Sequencing of the fragment confirmed the alternative splicing event from the canonical exon 41 to the alternative exon 42a (Figure S8). NCBI blast of the corresponding sequence demonstrated that the alternative exon 42a was located between exons 41 and 42 within the published *Lrrk2* sequence (Figure S8). To answer the question whether the expression is – like in case of skipped exon 5 – limited to certain cellular subtypes in the murine CNS, we analysed RNA samples from primary neuronal, microglia and astrocyte cultures and found identical patterns in samples from neurons and astrocytes (Figure 6E). Interestingly, no band was detectable in samples from microglia, indicating that these cells may not process transcripts containing the alternative exon 42a. For confirmation, we utilized a qPCR analysis using specific primers and probes for the full-length *Lrrk2* transcript (i.e. primers located in the endogenous exon 42) and primers and probes located in the alternative exon 42 (Figure 6G). As expected, in primary microglia we could barely detect the expression of the shortened *Lrrk2* mRNA transcript ending in the alternative exon 42, whereas primary neurons and astrocytes show significant higher expression of this variant.

Taken together, we were able to identify and describe a novel – and so far unknown – splice variant of *Lrrk2* (skipped exon 5) and confirm the presence of an already published alternative transcript (alternative exon 42a) in RNA samples from different brain regions, organs and primary neuronal cell cultures. In addition, we found differential expression of these new transcripts in a cell-type specific manner.

## Discussion

Several studies analyzing mRNA and protein expression of LRRK1 and LRRK2 in mouse, rat, pig and human samples of brain, organs/tissues and cells have been published so far, demonstrating largely ubiquitous expression of *Lrrk2* in the CNS and peripheral tissues [30], [31], [40]–[54]. Our present study presents novel findings, which gives potentially new insights into the physiological function of these proteins. To our knowledge, this is the first time that *Lrrk1* and *Lrrk2* expression was comparatively analysed, qualitatively and quantitatively, using several independent and highly sensitive analytical methods. With this we have thus described all important aspects like anatomical localization, expression level, distribution in neuronal cell populations and presence of splice variants in a single study.

We show a detailed analysis of *Lrrk1* and *Lrrk2* expression during embryonic development (E7.5 to E15.5) as well as in the postnatal (P0 to P21) and adult (>6 weeks) brain of C57BL/6J mice. For this purpose, we used radioactive labeled riboprobes, which are more sensitive than non-radioactive probes and thus are able to identify low expression sites [37]. In addition, we used up to 6 different overlapping and not overlapping riboprobes per gene (Figure S9), which all showed identical results after hybridization (data not shown). We also used the corresponding sense RNA probes as negative controls to ensure the specificity of the expression pattern. We found moderate ISH signals for *Lrrk1* and *Lrrk2* during embryogenesis (E10 to E12.5) with their expression patterns showing common characteristics in many organs and structures. Total level of *Lrrk1* were slightly stronger and more widespread than *Lrrk2*. With ongoing organogenesis (E15.5), the overall mRNA level of both genes increased and their non-neuronal expression domains became broader. Increasing *Lrrk2* expression during embryonic development was also described by Zechel *et al.*, who performed ISH with DIG-labeled riboprobes on brain sections from C57BL/6 mice and did not observe Lrrk2 expression in the CNS before embryonic stage E12.5 [54]. In addition, similar differences in total brain mRNA level of *Lrrk1* and *Lrrk2* were shown in two independent studies using radioactive ISH and qPCR [30], [31]. First, in rat embryos, Westerlund *et al.* determined postnatal day P8 to be the earliest time-point in which *Lrrk2* expression could be observed in the CNS, while widespread *Lrrk1* expression was found already at embryonic stage E 13.5 [31]. Secondly, Biskup *et al.* showed, in samples from whole mouse brains, that between embryonic stages E11.5 and E17.5 the relative mRNA level of *Lrrk1* were constantly higher than those of *Lrrk2* [30]. The fact that we and others – in contrast to our ISH studies - detected considerable level of *Lrrk1* and *Lrrk2* mRNA by RT-PCR or real-time PCR [30], [54] in samples of whole embryos and embryonic brain tissues, even at early stages of embryonic development, is not surprising. This can be explained by the observed strong expression of both genes in the choroid plexus and the meninges, which tightly surround the complete CNS and its ventricular system. In line with the non-neuronal expression of *Lrrk2* during development is the expression in the Rathke’s pouch; the non-neural origin of the adenohypophysis. Strikingly, in adulthood *Lrrk2* expression can be found in the anterior and intermediate lobe (adenohypophysis) and not in the posterior lobe (neurohypophysis) (data not shown), which is composed of cells of the ventral diencephalon [55]. Interestingly, there are also two groups presenting stronger ISH signals for *Lrrk1* in the developing CNS of rats [31] and for *Lrrk2* in the developing CNS of mice [54]. Nevertheless, even though these data may conflict with our findings, they could possibly originate from species or technical differences between their and our studies.

During embryonic development, we observed much stronger ISH signals for *Lrrk1* and *Lrrk2* expression in kidney, lung, liver and heart when compared to all CNS regions. This is fully in line with other comparative expression studies, most of which quantified mRNA level of different organs and brain regions by qPCR: (i) Zimprich *et al.* presented highest *Lrrk2* mRNA level in human lung [8]. (ii) Biskup *et al.* described that both *Lrrk1* and *Lrrk2* mRNA level were much stronger in murine neonatal lung, heart and kidney than in whole brain samples [30]. (iii) Larsen *et al.* demonstrated highest *Lrrk2* mRNA expression in porcine testis, spleen and lung [45]. (iv) Maekawa *et al.* showed highest *Lrrk2* mRNA level in kidney, spleen, lung and testis even in samples from adult (20, 50 and 98 week old) mice [46]. Taken together, *Lrrk1* and *Lrrk2* are widely expressed during whole embryogenesis in non-neuronal tissue whereas their expression in neural tissue is barely detectable. On the other hand, *Lrrk2* mutant mice (knockout, LRRK2 overexpression or introduction of point-mutations) do not show gross developmental phenotypes; they survive and show normal growth and life span [56]–[59]. This suggests that *Lrrk2* function during development is not necessary for survival, but is required for non-vital physiological function within non-neuronal tissues.

After birth, *Lrrk2* expression became robustly detectable in the murine CNS. It then increased further during early postnatal development in a highly dynamic manner and reached its maximum level around 6 weeks after birth, remaining stable throughout the lifespan. Thus, we confirmed the observations from other studies, namely ascending expression level of *Lrrk2* during different stages of postnatal development [31], [25], [30]. In the adult mouse brain, strong ubiquitous *Lrrk2* ISH signals were present, with highest level in striatum, cortex, hippocampus, cerebellum, anterior olfactory nucleus and brain stem. Interestingly, during these first postnatal weeks synaptogenesis reaches a peak, specifically in the striatum and cortex [60], [61]. This raises the questions whether *Lrrk2* is directly involved in synaptogenesis or is part of the synaptic machinery. Indeed, a synaptic function of Lrrk2 – as suggested by its appearance in postnatal stages – is also supported by the fact that Lrrk2 has been implicated in neurotransmitter release [36], [62]. In addition, LRRK2 protein expression becomes robustly detectable in primary hippocampal and cortical neuron-cultures (prepared from E15–16 embryonic brains) from day 7 onwards: a time point when the neuronal networks in the cultures are established [36]. Surprisingly, the mesencephalic region of the substantia nigra (SN), which contains the most vulnerable neuronal populations affected in PD, exhibited only moderate *Lrrk2* expression in the adult brain: the target area of these dopaminergic neurons (i.e. caudate putamen in the striatum) shows the highest level of *Lrrk2* mRNA. Most likely due to the rather low expression level of *Lrrk2* in the SNc, there have been conflicting statements concerning *Lrrk2* expression in this region. Some groups present only low to undetectable level of *Lrrk2* in the SNc of mice [47], [53] and rats [41], [53]. On the other hand, others found clearly detectable *Lrrk2* expression in the SNc of humans [40], [43], [51], mice [44], [49], [52] and rats [40], [42]. Still, the combination of these and the present study clearly indicates that *Lrrk2* is indeed expressed in the adult SNc.

In contrast to *Lrrk2*, *Lrrk1* was barely detectable in the adult mouse brain and only present in the mitral cell layer of the olfactory bulb. It is quite surprising that, despite their high degree of homology and their comparable expression patterns during development, the adult brain almost exclusively expresses *Lrrk2*. Nevetheless, this finding suggests that LRRK1 does not compensate for LRRK2 function in the adult brain and – as a consequence – is not responsible for the absence of neurodegeneration in genetic animal models of *Lrrk2* [62]. Furthermore it may also explain why, up to now, no PD-associated mutation has been identified in *LRRK1* [28].

We further verified our observed ISH-based expression pattern of *Lrrk2* by exact quantification of mRNA and protein level in samples from different brain regions using real-time PCR and Western blot, respectively. The observed patterns match remarkably, not only with the strongest *Lrrk2* expression domains identified by our ISH analyses, but also with Northern blot [7], real-time PCR [48], [53], [63] and Western blot data [49] published by other groups. Interestingly, apart from striatum, no discrepancies between the relative protein and mRNA level in the different brain regions could be detected, suggesting no major posttranscriptional regulation of *Lrrk2* outside of the striatum. The discrepancy within the striatum could have manifold explanations including (i) posttranscriptional regulation of *Lrrk2* in the striatum via specific microRNAs, leading to blocking of translation [64], (ii) reduced half-life of LRRK2 protein in the striatum due to protein truncations, protein degradation or diminished protein stabilization, (iii) the opposite scenario – increased half-life of the LRRK2 protein in the striatum, so that less mRNA transcripts would be required to maintain the necessary protein level, or (iv) transport of the protein from the striatum to other brain compartments. Thus, further analyses are necessary to distinguish between these theories.

In the next part of the study, *Lrrk2* expression was analysed on the cellular level in sections from striatum and cortex with focus on dopaminoceptive neurons. Elucidation of neuronal versus non-neuronal (i.e. the role of *Lrrk2* in microglia function as mediator of neuroinflammation) localization of LRRK2 could help to understand its physiological function and/or its role in the pathogenesis of *Lrrk2*-associated PD [65]–[67]. Indeed, our studies also confirmed expression of *Lrrk2* in glial cells; however, the bulk of the expression in the CNS was neuronal. In addition, almost 100% of the *Lrrk2*+ cells in the striatum were positive for the MSN-marker DARPP-32, indicating that Lrrk2 might play a role in dopaminoceptive neurons. Quantification of double ISH/IHC in the striatum revealed that about 61% (36% *Lrrk2*+, 25% DRD1a+, 39% *Lrrk2*+ and DRD1a+) of DRD1a+ MSNs, representing the direct pathway, highly express *Lrrk2* mRNA. In this regard, MSNs of the indirect pathway (DRD2+) exhibited almost identical percentages (38% *Lrrk2*+, 24% DRD2+, 37% *Lrrk2*+ and DRD2+), indicating that only subpopulations of both pathways express high level of LRRK2. This notion was supported by a partial colocalization of Lrrk2 with DRD1a+ and DRD2+ neurons in the striatum. These data confirm the study of Mandemakers *et al.* where, via double IHC, LRRK2 protein was shown to colocalize with DRD1a in striatal cells [47] on the mRNA level. Our quantitative data also reveal that only subpopulations of both the direct and the indirect pathway express very high level of LRRK2. Thus, we hypothesize that *Lrrk2* could play a role in both dopaminergic pathways, which are directly related to the neuropathology of PD [68]. Nevertheless, differential function(s) of *Lrrk2* in DRD1a positive and in DRD2 positive subpopulations of neurons has to be evaluated.

Next, we concentrated on the search for splice variants of the murine *Lrrk2* mRNA transcript in various brain regions and organs from adult mice. Zimprich *et al.* [8] (2004) presented the very first hints for alternative *Lrrk2* splicing, when they noticed weak bands at lower sizes in Northern blot analyses of human brain samples; this was also observed by an independent group [7]. When they used RT-PCR to quantify transcripts containing exons 1–3, 12–13 and 32–34 however, they did not find significant differences in the expression level [8]. Later, West *et al.* described the presence of at least six transcription start sites upstream of the first Kozak sequence and one downstream of the first predicted exon (within intron 1). This would exclude the complete *Lrrk2* ORF and produce a protein of unknown biological significance [69]. However, they also found no evidence for alternate *Lrrk2* exons or protein coding sequences [69]. Therefore, we analysed the comparably large *Lrrk2* transcript for exons, which could be spliced out without causing dramatic rearrangements, and performed RT-PCR analyses using primer pairs amplifying the regions of exons 4–9, 16–21, 30–34, 35–39, 41–45 and 47–51. After screening these six different regions, we were able to identify a novel – and so far unknown – splice variant of *Lrrk2*, sparing exon 5 (135bp).

Ignacio Marin has published, in 2008, the detailed structure of selected LRRK proteins, including human LRRK2, and described the presence of 14 N-terminal LRRK2-specific repeats in the human and mouse sequence [21]. We calculated that exon 5 covers virtually the complete sequence of the fourth repeat. The lack of exon 5 (and consequently the loss of the fourth repeat) may result in a protein with altered size (45 amino acids smaller) and unknown biological significance. Nevertheless, a possible consequence could be a change in the affinity to different LRRK2 binding partners (i.e. alterations in the LRRK2 interactome). Interestingly, this alternative transcript was present in all samples analysed. When we analyzed RNA samples from primary neuronal, microglia and astrocyte cultures (all prepared from cortical tissues) however, we found identical patterns in neurons and microglia. Surprisingly, there was a dominant expression of the splice variant without exon 5 in astrocytes. Thus, astrocytes may exhibit a different interactome of LRRK2 and therefore LRRK2 function may differ in neurons and astrocytes. Furthermore, we were able to confirm the presence of an alternative transcript that contains an alternative exon 42a (including several stop codons) and was located between the endogenous exons 41 and 42 (annotated in ensembl.org). Interestingly, we also found cell-type specific expression patterns of these transcripts between the different primary cell cultures. While neurons and astrocytes both process the transcript containing the alternative exon 42a, no band was visible in microglia. This suggests a rather low expression of the alternative variant compared to the full-length *Lrrk2* transcript. As mentioned above, we have no information about the sequence that is 5′ of exon 39 in this transcript. Assuming that the transcript starts regularly with exon 1, it could induce a maximum protein product of 239 kDa (in contrast to the 280 kDa full-length LRRK2 protein). This could somehow explain why we (data not shown) and others [30], [46], [47], [50], [65], [70], [71] observe additional bands in Western blot analyses, which could fit to the calculated size of a truncated LRRK2 isoform. Considering the complex architecture of the LRRK2 protein, implying that *Lrrk2* is involved in more than one cellular process [72], we found that the endogenous exons 41, 42 and 43 cover the complete end of the MAPKKK domain [73]. Splicing that introduces an alternative exon 42a will lead to an early truncation of the protein (due to its included stop codons, and may seriously influence its enzymatic activity (due to lack of the c-terminal part of the kinase domain). In addition, the complete WD40 domain would also be absent in such a truncation. Jorgensen *et al.* demonstrated that removing the WD40 domain prevents complex formation and autophosphorylation but also blocks the neurotoxicity of LRRK2 PD mutations [74]. Moreover, it was shown that fragments of LRRK2 containing the ROCO domain mediate LRRK2 kinase inhibition [75]. It is thus tempting to speculate that the splice variant with alternative exon 42a is acting as an inhibitor of intrinsic LRRK2 function. The absence of this variant in microglia would, in turn, render this cell type more susceptible to LRRK2 pathogenic mutations via further stimulation or through missing the regulation of its potentially toxic kinase activity. It is interesting to note that attenuated inflammatory responses after knockdown of LRRK2 in microglia indicate that it may be a positive regulator of neuroinflammation [67]. Taken together, we observed cell-type specific differences in the expression of full-length, exon 5-skipped and truncated (E1–E42a) transcripts. Thus, we assume that *Lrrk2* features different physiological functions in different neural cell populations and cell-type specific differences in its enzymatic functions and its interactome. This is in full agreement with Lewis *et al*. [72], who postulated that alterations in different glial cell lines could selectively affect susceptible neuronal populations in PD. Furthermore, *Lrrk2* may be regulated by different signals; interacting with different upstream regulators or downstream effectors: phosphorylating different substrates, and so form – depending on the presence of appropriate cofactors, interacting proteins or other favorable environmental conditions – tissue-selective or non-selective signaling complexes. This results in cell-type specific molecular effects and pathology [72]. Further efforts are necessary to figure out if these splicing patterns are conserved between mouse and humans, or if they represent interesting differences between species.

In conclusion, we present here a substantial and detailed analysis of *Lrrk1* and *Lrrk2* expression, focusing on qualitative and quantitative aspects (anatomical localization, expression level, distribution in neuronal cell populations and presence of splice variants) and covering numerous stages ranging from ES cells, embryonic development until the postnatal and adult brain. To our knowledge, we show for the first time a quantitative analysis of colocalization between *Lrrk2* mRNA and both DRD1a and DRD2 expression in MSNs of the striatum. In addition, we were able to identify a novel splice variant of *Lrrk2* (skipped exon 5) and confirm an annotated transcript (alternative exon 42a) in RNA samples from different brain regions, organs and primary neuronal cell cultures. Besides that, we found cell-type specific alterations in the transcript expression patterns in the different neuronal lineages.

## Supporting Information

Figure S1 **Expression analysis of** ***Lrrk2*** **mRNA in early- to midgestation embryos by RT-PCR and** ***in situ*** **hybridization (ISH).** **(A)** RNA samples from ES cells and early embryos (E8.5 to E12.5), as well as newborn mice (P0) and adult kidney, which served as positive controls, were analysed by RT-PCR for *Lrrk2* expression. NoRT, negative control. **(B)** ISH for *Lrrk2* mRNA in sections from E7.5 embryos. Depicted is a brightfield image (B) for anatomical orientation and a darkfield image (B') showing the ISH signals in white). Note the strong mRNA signal in the parietal yolk sac (arrowhead) and the decidua (arrow). **(C–E)** ISH for *Lrrk2* mRNA in sections from E14.5 embryos. The overview sagittal section exhibits strong expression in the choroid plexus (Cp), in chondral structures of the nasal capsule and the kidney (Ki) **(C)**. Detailed view of the lower jar reveales strong *Lrrk2* expression in the primordium of upper and lower incisive tooth **(D)**. Detailed view of the developing kidney with strong expression in the metanephric vesicles (arrows) **(E)**. Abbreviations: Al, allantois; Ch, chorion; Cp, choroid plexus; Cx, cortex; Dc, decidua; Df, forelimb digit; Ee, embryonic ectoderm; Em, embryonic mesoderm; En, embryonic endoderm; Ex, exocoelomic cavity; Id, intervertebral disc; In, incisive; Ki, kidney; Li, liver; Lj, lower jar; Lu, lung; Py, parietal yolk sac; To, tongue. Scale bars represent 200 µm in B, 2 mm in C, 250 µm in D–E.
(TIF)

Figure S2 **Expression analysis of** ***Lrrk2*** **mRNA in the postnatal mouse brain.** ISH for *Lrrk2* mRNA in sections from P0 and P7 mice. For each brain, a brightfield image (left image, for anatomical orientation) and a darkfield image (right image, ISH signals in white) are shown. **(A)** Weak *Lrrk2* expression is detected in the murine CNS for the first time directly after birth (in P0 sections), with signals present in the developing cortex (Cx), cerebellum (Cb) and brainstem (Bs). Strong *Lrrk2* mRNA expression can be detected in the choroid plexus (white arrows in A′). **(B)** At stage P7, *Lrrk2* mRNA level in forebrain structures like cortex, striatum (St) and olfactory bulb (Ob) further increases. Note that the strong signals in the choroid plexus (white arrows in B′) persist throughout development. Abbreviations: Bs, brain stem; Cb, cerebellum; Cp, choroid plexus; Cx, cortex; Hi, hippocampus; Ht, hypothalamus; Mb, midbrain; Ob, olfactory bulb; St, striatum; Ta, Thalamus. Scale bars represent 2 mm.
(TIF)

Figure S3 **Expression analysis of** ***Lrrk1*** **and** ***Lrrk2*** **mRNA in the adult visual cortex.** ISH for *Lrrk1* **(A)** and *Lrrk2* **(B)** mRNA in sagital sections from adult mice. As a negative control **(C)**, a sense probe has been used. Note the strong expression of *Lrrk2* mRNA in the cortical layers 2 to 5a, whereas ISH for *Lrrk1* mRNA only depicts background staining comparable to the negative control. Abbreviations: I to VIb indicate the different cortical layers. Scale bars represent 200 µm.
(TIF)

Figure S4 **Microautoradigraphy of** ***Lrrk1*** **and** ***Lrrk2*** **mRNA in different regions of the adult mouse brain.** High magnification brightfield images depicting positive ISH signal for *Lrrk1* or *Lrrk2* mRNA as condensed black granules: **(A–B)** Highest level of *Lrrk2* expression in the murine CNS is detected in the striatum. No *Lrrk1* signal can be detected. **(C–D)** The subventricular zone (SVZ) is showing *Lrrk2* but not *Lrrk1* expression both in ependymal cell as well as in deeper layers of the SVZ (arrows in D). Note also the strong Lrrk2 expression in the choroid plexus. **(E–F)** The pyramidal cells hippocampus proper show the highest level *Lrrk2* mRNA expression in this region. Nevertheless also other regions show positive signal. ISH for *Lrrk1* only depicts background signal. **(G–H)** In the substantia nigra *Lrrk2* expression can be found predominantly in the pars compacta (arrows in H). Again *Lrrk1* mRNA could not be detected. **(I–J)** In cerebellum, strongest *Lrrk2* expression can be found in the purkinje cells (arrows in J). Significant *Lrrk1* expression is limited to the meninges (arrows in I). Abbreviations: CA3, CA3 region of the hippocampus proper; Cp, choroid plexus; DG, dentate gyrus; Ec, ependymal cells; GL, granular layer of the cerebellum; Hi, hippocampus; Ht, hypothalamus; LV, lateral ventricle; Me, meninges; ML, molecular layer of the cerebellum; PC, purkinje cell layer; SNpc, substantia nigra pars compacta; SNpr, substantia nigra pars reticulata; SVZ, subventricular zone; Ta, Thalamus. Scale bars represent 50 µm.
(TIF)

Figure S5 **Qualitative expression analysis of** ***Lrrk2*** **mRNA transcripts.** **(A)** Schematic overview of the *Lrrk2* genomic sequence and location of the 51 exons, which code for the full-length *Lrrk2* mRNA. Exons with a length (number of base-pairs) that is divisible by three are highlighted in green (i.e. these exons could theoretically be skipped without resulting in a frame-shift of the residual mRNA coding sequence). Areas of the *Lrrk2* gene which have been analysed by RT-PCR are indicated above. Sequence is based on the NCBI accession number NW_001030577.1. **(B–F)** RNA samples from different brain regions and organs were analysed by RT-PCR using primers that amplify *Lrrk2* transcripts between exons 16 and 21 **(B)**, exons 30 and 34 **(C)**, exons 35 and 39 **(D)**, exons 41 and 45 **(E)**, and exons 47 and 51 **(F)**. Note that there are no additional bands visible in all samples analysed.
(TIF)

Figure S6 **Sequence analysis of the novel** ***Lrrk2*** **splice variant lacking exon 5. (A)** Sequencing chromatogram of the boundary region between exons 4 and 6 indicating the skipping of exon 5. **(B)** The sequence of the endogenous *Lrrk2* (top row) was aligned to the sequence of the alternative processed transcript with skipped exon 5 (bottom row). The resulting protein sequence and positions of the LRRK2-specific repeats (underlined amino acids) are indicated below. **(C)** Schematic overview of the endogenous LRRK2 protein structure (top) versus the putative protein product lacking exon 5 (deleted LRRK2-specific repeat indicated in red).
(TIF)

Figure S7 **Schematic overview of the endogenous** ***Lrrk2*** **and an alternative processed transcript. (A)** The full length *Lrrk2* transcript consisting of 51 exons, 8275 base-pairs and 2527 amino acids (according to ensembl.org sequence ENSMUST00000060642) is shown on top. **(B)** An alternative processed transcript annotates 19 of these exons and 3452 base-pairs (according to ensembl.org sequence ENSMUST00000140734, shown on bottom). Note that the alternative processed transcript contains an alternative exon 42a, which is not present in the endogenous *Lrrk2* transcript.
(TIF)

Figure S8 **Sequence analysis of the** ***Lrrk2*** **splice containing an additional exon 42. (A)** Sequencing chromatogram of the boundary region between endogenous exon 41 and alternative exon 42 (indicating the alternative splicing event). **(B)** The sequence of the endogenous *Lrrk2* (top row) was aligned to the sequence of the alternative processed transcript that contains an alternative exon 42a (bottom row). The resulting protein sequence is indicated below. **(D)** Schematic overview of the endogenous LRRK2 protein structure (top) versus the putative protein product which is truncated after exon 42a (bottom). Note that the putative protein is truncated within the MAPKKK domain and there is no information about the N-terminal part (shaded).
(TIF)

Figure S9 **Sequence of primers and probes used in this study.** Probes for qPCR were all 5′-labeled with 6-carboxyfluorescin (FAM) and 3′-labeled with Black Hole Quencher (BHQ-1). LNA-bases are indicated by bold letters.
(TIF)
