# Supplementary figures and images for: Expression Analysis of Lrrk1, Lrrk2 and Lrrk2 Splice Variants in Mice
Source: PLoS One. 2013 May 10;8(5):e63778. doi: 10.1371/journal.pone.0063778 (PMC3651128; doi:10.1371/journal.pone.0063778)

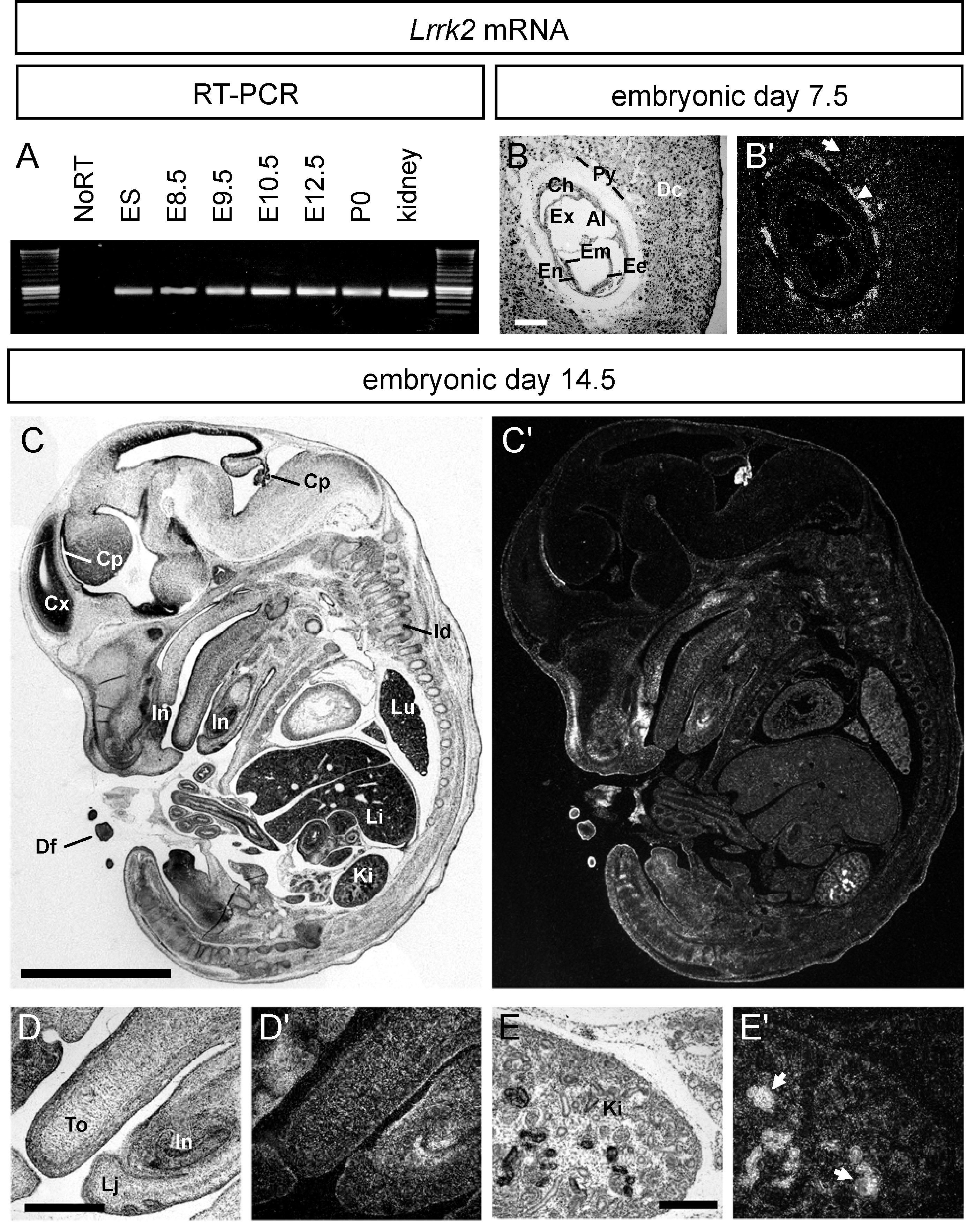

Supplement: Figure S1 — Expression analysis of Lrrk2 mRNA in early- to midgestation embryos by RT-PCR and in situ hybridization (ISH). (A) RNA samples from ES cells and early embryos (E8.5 to E12.5), as well as newborn mice (P0) and adult kidney, which served as positive controls, were analysed by RT-PCR for Lrrk2 expression. NoRT, negative control. (B) ISH for Lrrk2 mRNA in sections from E7.5 embryos. Depicted is a brightfield image (B) for anatomical orientation and a darkfield image (B') showing the ISH signals in white). Note the strong mRNA signal in the parietal yolk sac (arrowhead) and the decidua (arrow). (C–E) ISH for Lrrk2 mRNA in sections from E14.5 embryos. The overview sagittal section exhibits strong expression in the choroid plexus (Cp), in chondral structures of the nasal capsule and the kidney (Ki) (C). Detailed view of the lower jar reveales strong Lrrk2 expression in the primordium of upper and lower incisive tooth (D). Detailed view of the developing kidney with strong expression in the metanephric vesicles (arrows) (E). Abbreviations: Al, allantois; Ch, chorion; Cp, choroid plexus; Cx, cortex; Dc, decidua; Df, forelimb digit; Ee, embryonic ectoderm; Em, embryonic mesoderm; En, embryonic endoderm; Ex, exocoelomic cavity; Id, intervertebral disc; In, incisive; Ki, kidney; Li, liver; Lj, lower jar; Lu, lung; Py, parietal yolk sac; To, tongue. Scale bars represent 200 µm in B, 2 mm in C, 250 µm in D–E. (TIF) [file pone.0063778.s001.tif]

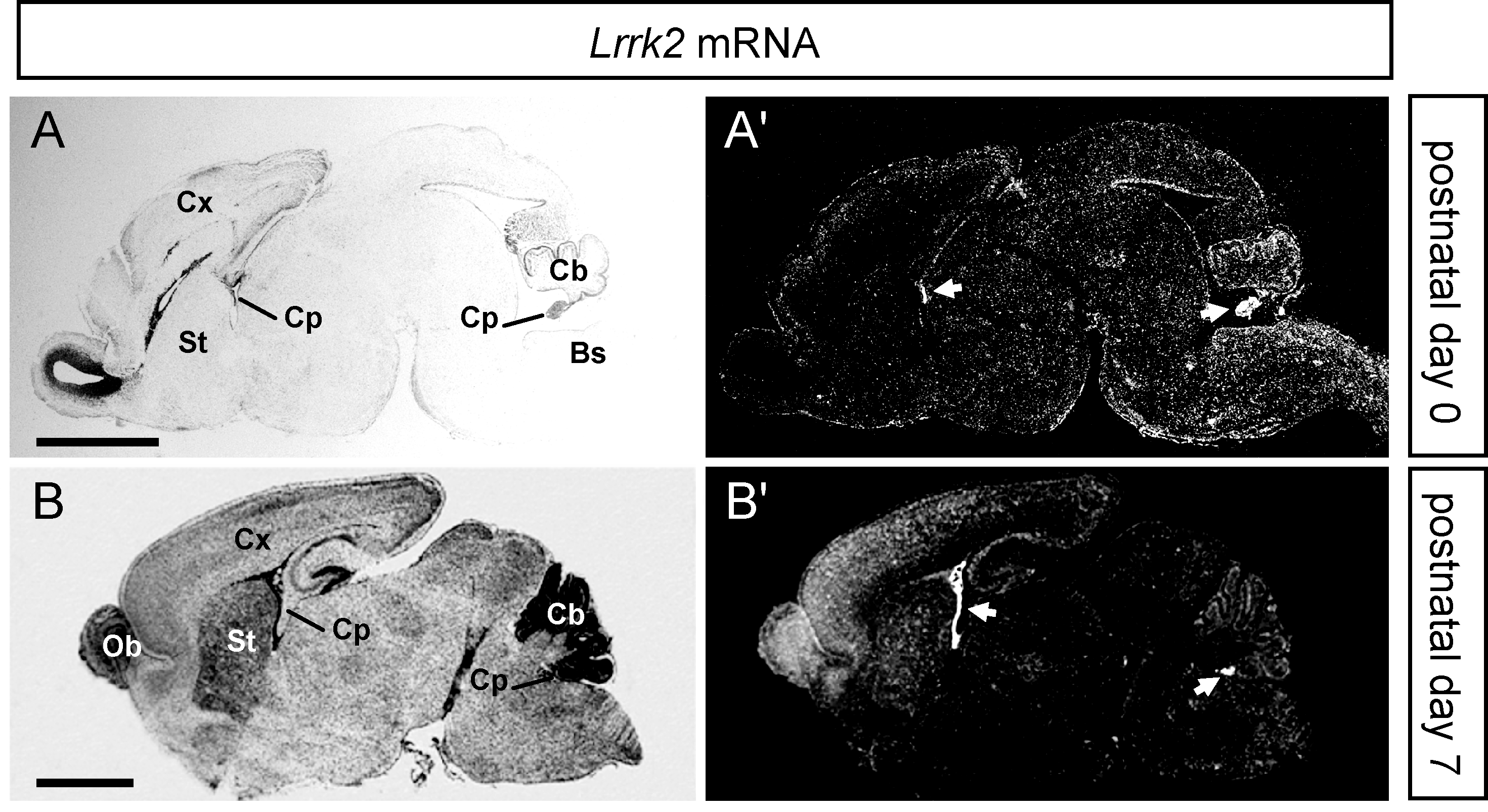

Supplement: Figure S2 — Expression analysis of Lrrk2 mRNA in the postnatal mouse brain. ISH for Lrrk2 mRNA in sections from P0 and P7 mice. For each brain, a brightfield image (left image, for anatomical orientation) and a darkfield image (right image, ISH signals in white) are shown. (A) Weak Lrrk2 expression is detected in the murine CNS for the first time directly after birth (in P0 sections), with signals present in the developing cortex (Cx), cerebellum (Cb) and brainstem (Bs). Strong Lrrk2 mRNA expression can be detected in the choroid plexus (white arrows in A′). (B) At stage P7, Lrrk2 mRNA level in forebrain structures like cortex, striatum (St) and olfactory bulb (Ob) further increases. Note that the strong signals in the choroid plexus (white arrows in B′) persist throughout development. Abbreviations: Bs, brain stem; Cb, cerebellum; Cp, choroid plexus; Cx, cortex; Hi, hippocampus; Ht, hypothalamus; Mb, midbrain; Ob, olfactory bulb; St, striatum; Ta, Thalamus. Scale bars represent 2 mm. (TIF) [file pone.0063778.s002.tif]

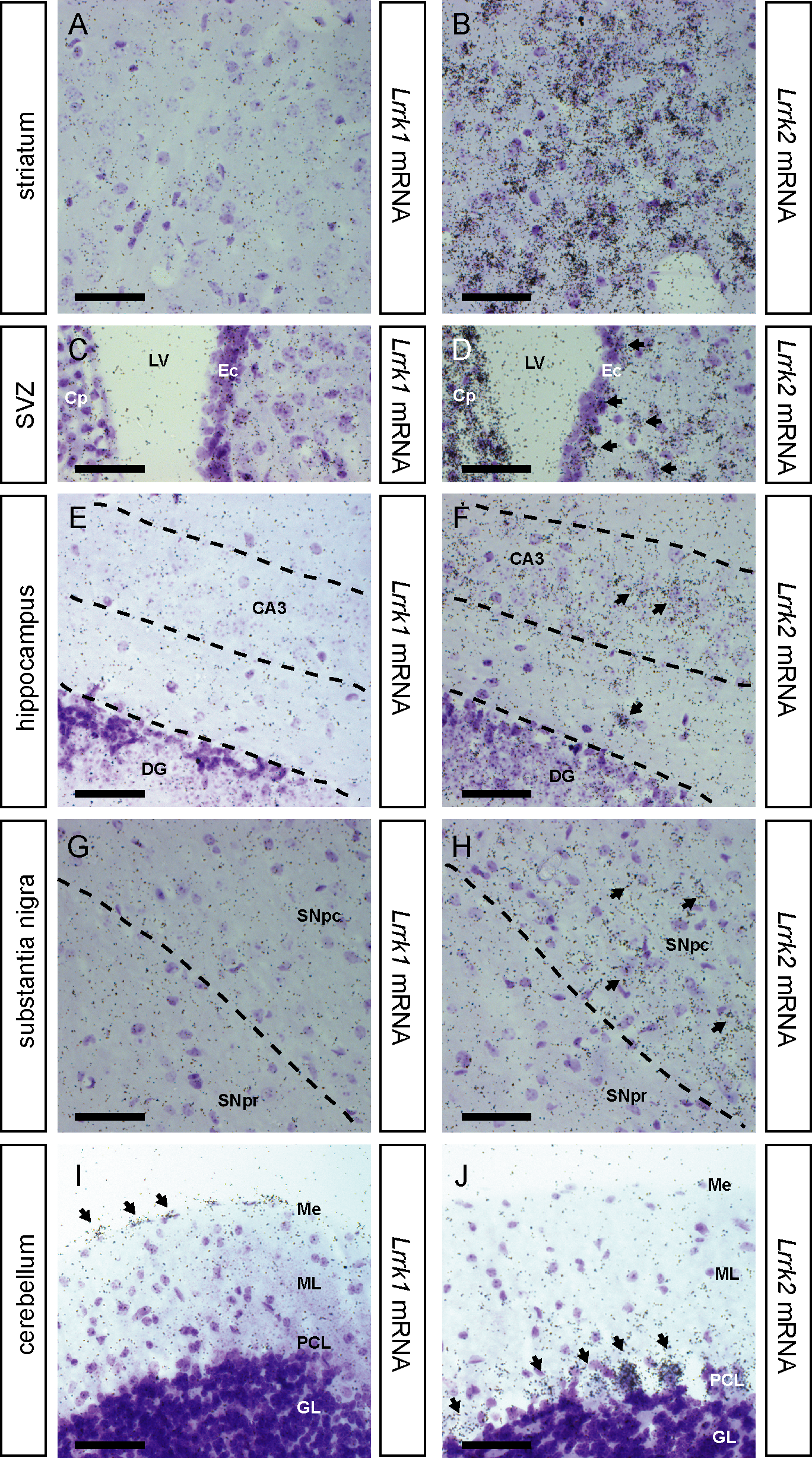

Supplement: Figure S3 — Expression analysis of Lrrk1 and Lrrk2 mRNA in the adult visual cortex. ISH for Lrrk1 (A) and Lrrk2 (B) mRNA in sagital sections from adult mice. As a negative control (C), a sense probe has been used. Note the strong expression of Lrrk2 mRNA in the cortical layers 2 to 5a, whereas ISH for Lrrk1 mRNA only depicts background staining comparable to the negative control. Abbreviations: I to VIb indicate the different cortical layers. Scale bars represent 200 µm. (TIF) [file pone.0063778.s003.tif]

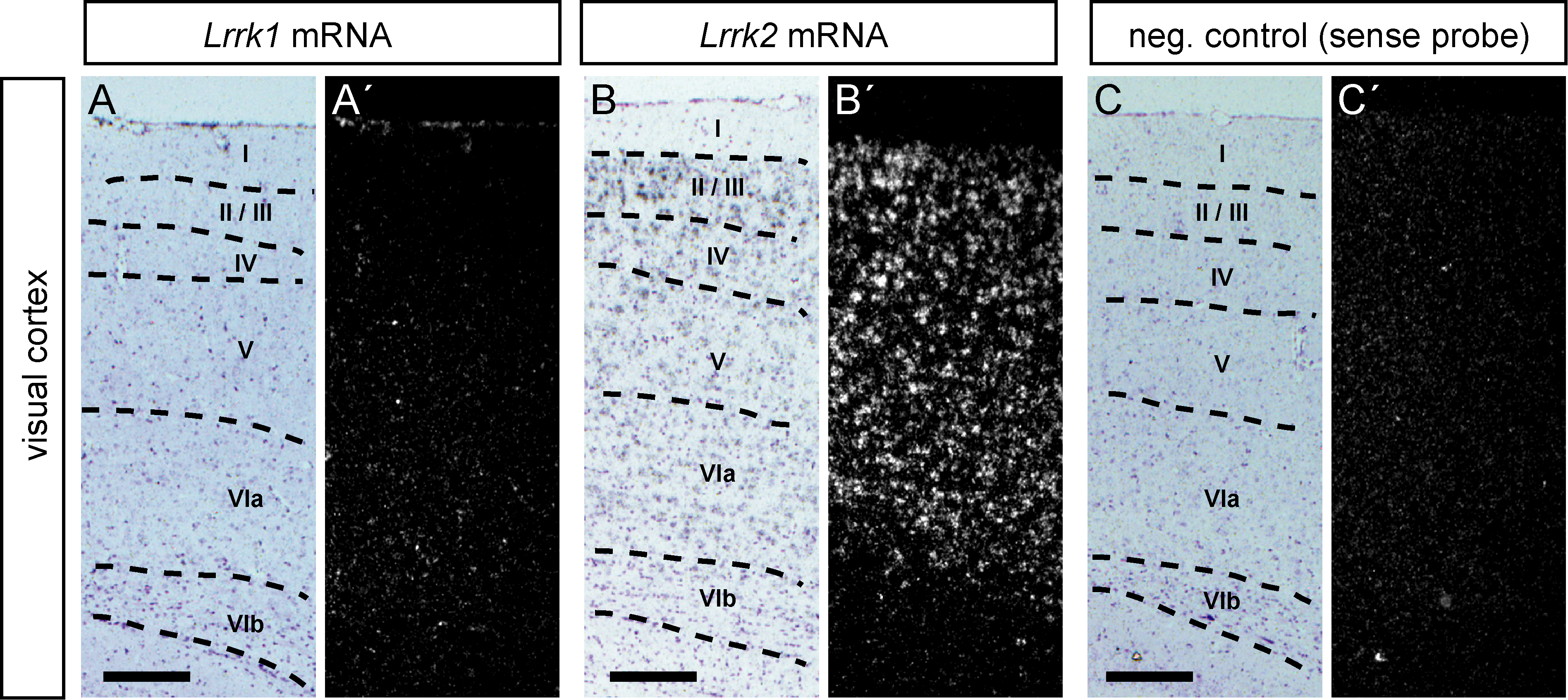

Supplement: Figure S4 — Microautoradigraphy of Lrrk1 and Lrrk2 mRNA in different regions of the adult mouse brain. High magnification brightfield images depicting positive ISH signal for Lrrk1 or Lrrk2 mRNA as condensed black granules: (A–B) Highest level of Lrrk2 expression in the murine CNS is detected in the striatum. No Lrrk1 signal can be detected. (C–D) The subventricular zone (SVZ) is showing Lrrk2 but not Lrrk1 expression both in ependymal cell as well as in deeper layers of the SVZ (arrows in D). Note also the strong Lrrk2 expression in the choroid plexus. (E–F) The pyramidal cells hippocampus proper show the highest level Lrrk2 mRNA expression in this region. Nevertheless also other regions show positive signal. ISH for Lrrk1 only depicts background signal. (G–H) In the substantia nigra Lrrk2 expression can be found predominantly in the pars compacta (arrows in H). Again Lrrk1 mRNA could not be detected. (I–J) In cerebellum, strongest Lrrk2 expression can be found in the purkinje cells (arrows in J). Significant Lrrk1 expression is limited to the meninges (arrows in I). Abbreviations: CA3, CA3 region of the hippocampus proper; Cp, choroid plexus; DG, dentate gyrus; Ec, ependymal cells; GL, granular layer of the cerebellum; Hi, hippocampus; Ht, hypothalamus; LV, lateral ventricle; Me, meninges; ML, molecular layer of the cerebellum; PC, purkinje cell layer; SNpc, substantia nigra pars compacta; SNpr, substantia nigra pars reticulata; SVZ, subventricular zone; Ta, Thalamus. Scale bars represent 50 µm. (TIF) [file pone.0063778.s004.tif]

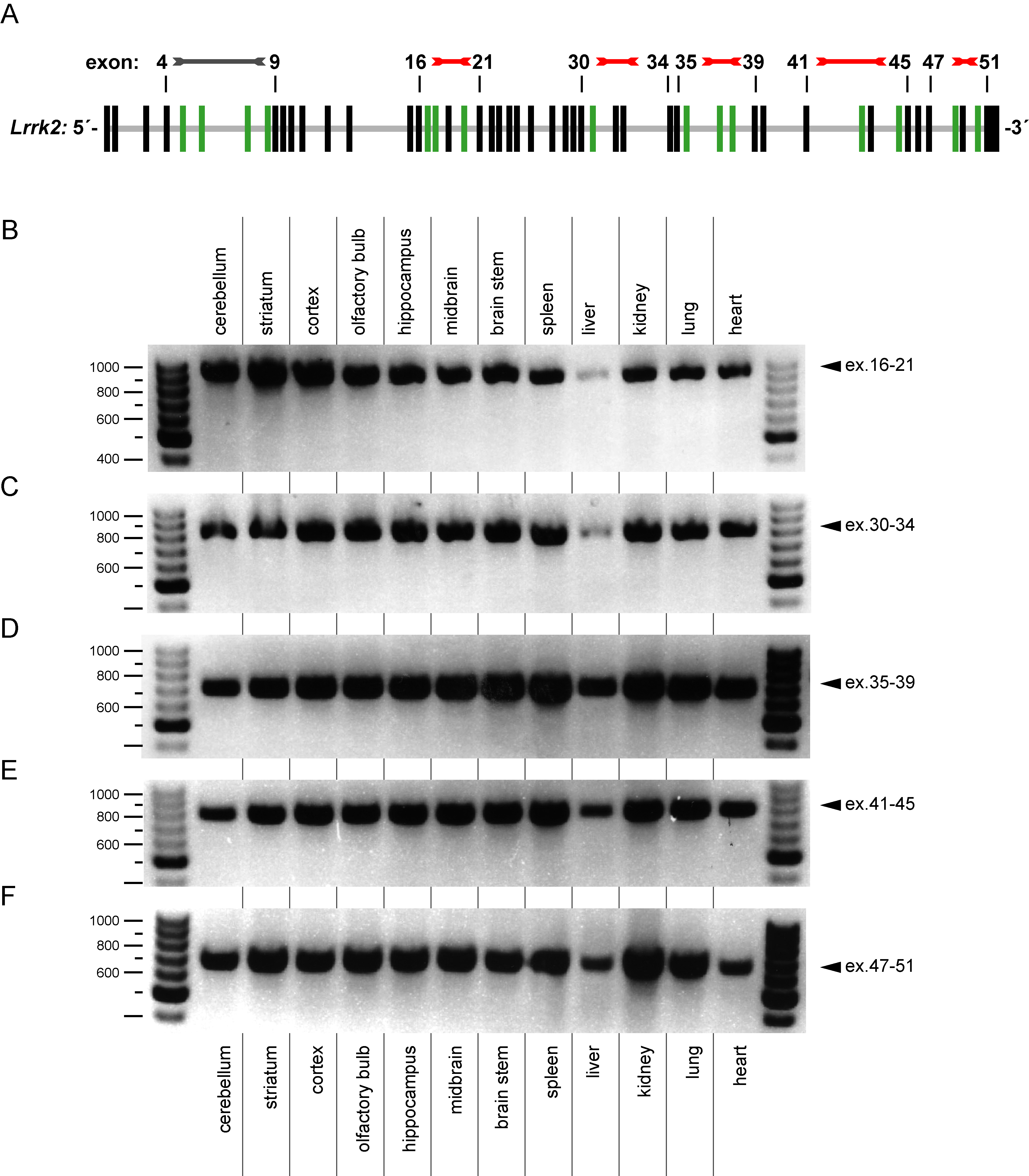

Supplement: Figure S5 — Qualitative expression analysis of Lrrk2 mRNA transcripts. (A) Schematic overview of the Lrrk2 genomic sequence and location of the 51 exons, which code for the full-length Lrrk2 mRNA. Exons with a length (number of base-pairs) that is divisible by three are highlighted in green (i.e. these exons could theoretically be skipped without resulting in a frame-shift of the residual mRNA coding sequence). Areas of the Lrrk2 gene which have been analysed by RT-PCR are indicated above. Sequence is based on the NCBI accession number NW_001030577.1. (B–F) RNA samples from different brain regions and organs were analysed by RT-PCR using primers that amplify Lrrk2 transcripts between exons 16 and 21 (B), exons 30 and 34 (C), exons 35 and 39 (D), exons 41 and 45 (E), and exons 47 and 51 (F). Note that there are no additional bands visible in all samples analysed. (TIF) [file pone.0063778.s005.tif]

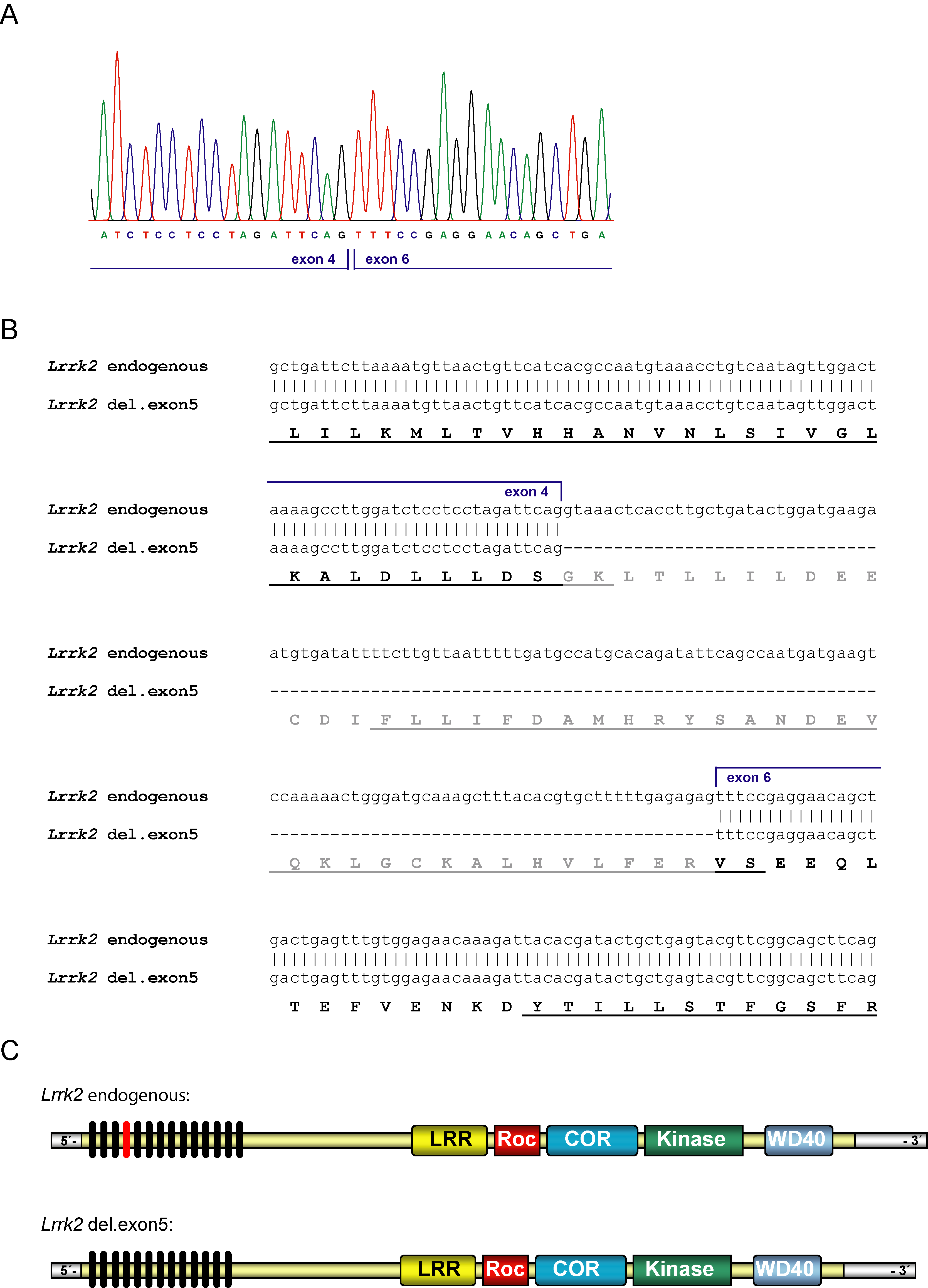

Supplement: Figure S6 — Sequence analysis of the novel Lrrk2 splice variant lacking exon 5. (A) Sequencing chromatogram of the boundary region between exons 4 and 6 indicating the skipping of exon 5. (B) The sequence of the endogenous Lrrk2 (top row) was aligned to the sequence of the alternative processed transcript with skipped exon 5 (bottom row). The resulting protein sequence and positions of the LRRK2-specific repeats (underlined amino acids) are indicated below. (C) Schematic overview of the endogenous LRRK2 protein structure (top) versus the putative protein product lacking exon 5 (deleted LRRK2-specific repeat indicated in red). (TIF) [file pone.0063778.s006.tif]

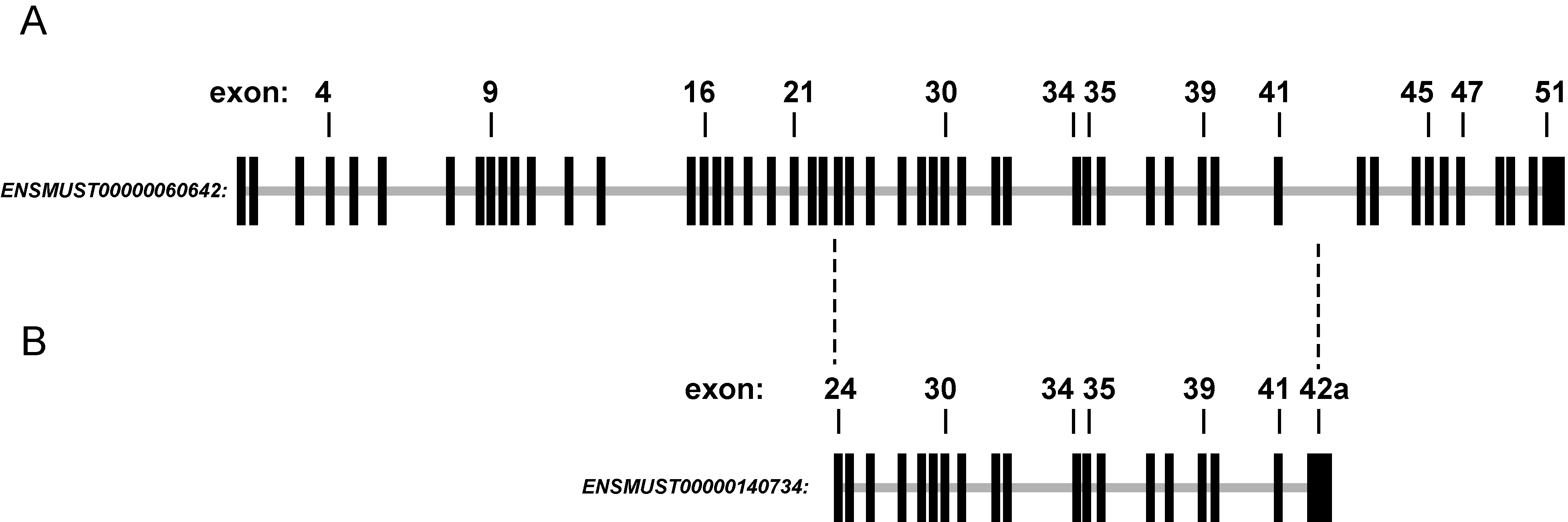

Supplement: Figure S7 — Schematic overview of the endogenous Lrrk2 and an alternative processed transcript. (A) The full length Lrrk2 transcript consisting of 51 exons, 8275 base-pairs and 2527 amino acids (according to ensembl.org sequence ENSMUST00000060642) is shown on top. (B) An alternative processed transcript annotates 19 of these exons and 3452 base-pairs (according to ensembl.org sequence ENSMUST00000140734, shown on bottom). Note that the alternative processed transcript contains an alternative exon 42a, which is not present in the endogenous Lrrk2 transcript. (TIF) [file pone.0063778.s007.tif]

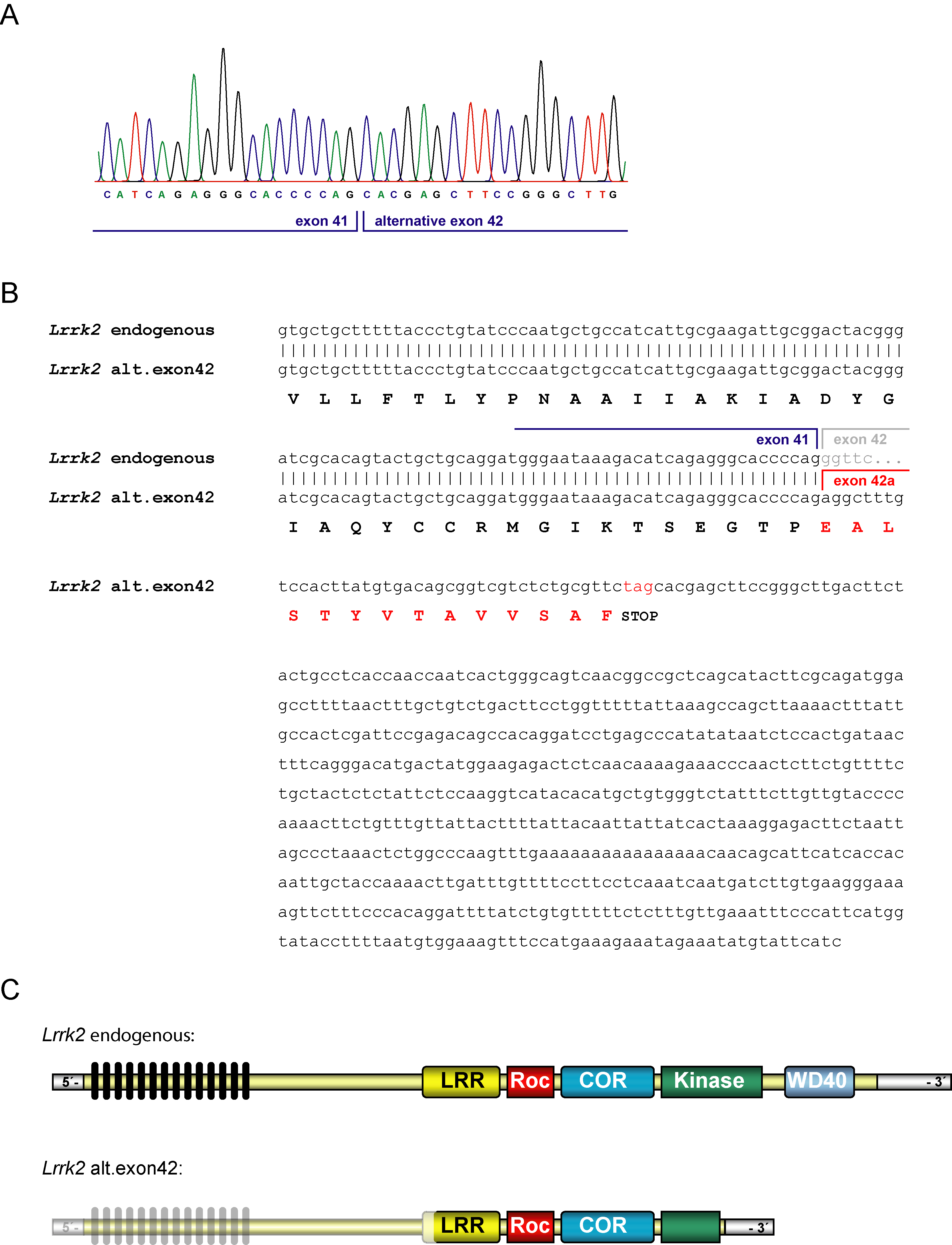

Supplement: Figure S8 — Sequence analysis of the Lrrk2 splice containing an additional exon 42. (A) Sequencing chromatogram of the boundary region between endogenous exon 41 and alternative exon 42 (indicating the alternative splicing event). (B) The sequence of the endogenous Lrrk2 (top row) was aligned to the sequence of the alternative processed transcript that contains an alternative exon 42a (bottom row). The resulting protein sequence is indicated below. (D) Schematic overview of the endogenous LRRK2 protein structure (top) versus the putative protein product which is truncated after exon 42a (bottom). Note that the putative protein is truncated within the MAPKKK domain and there is no information about the N-terminal part (shaded). (TIF) [file pone.0063778.s008.tif]

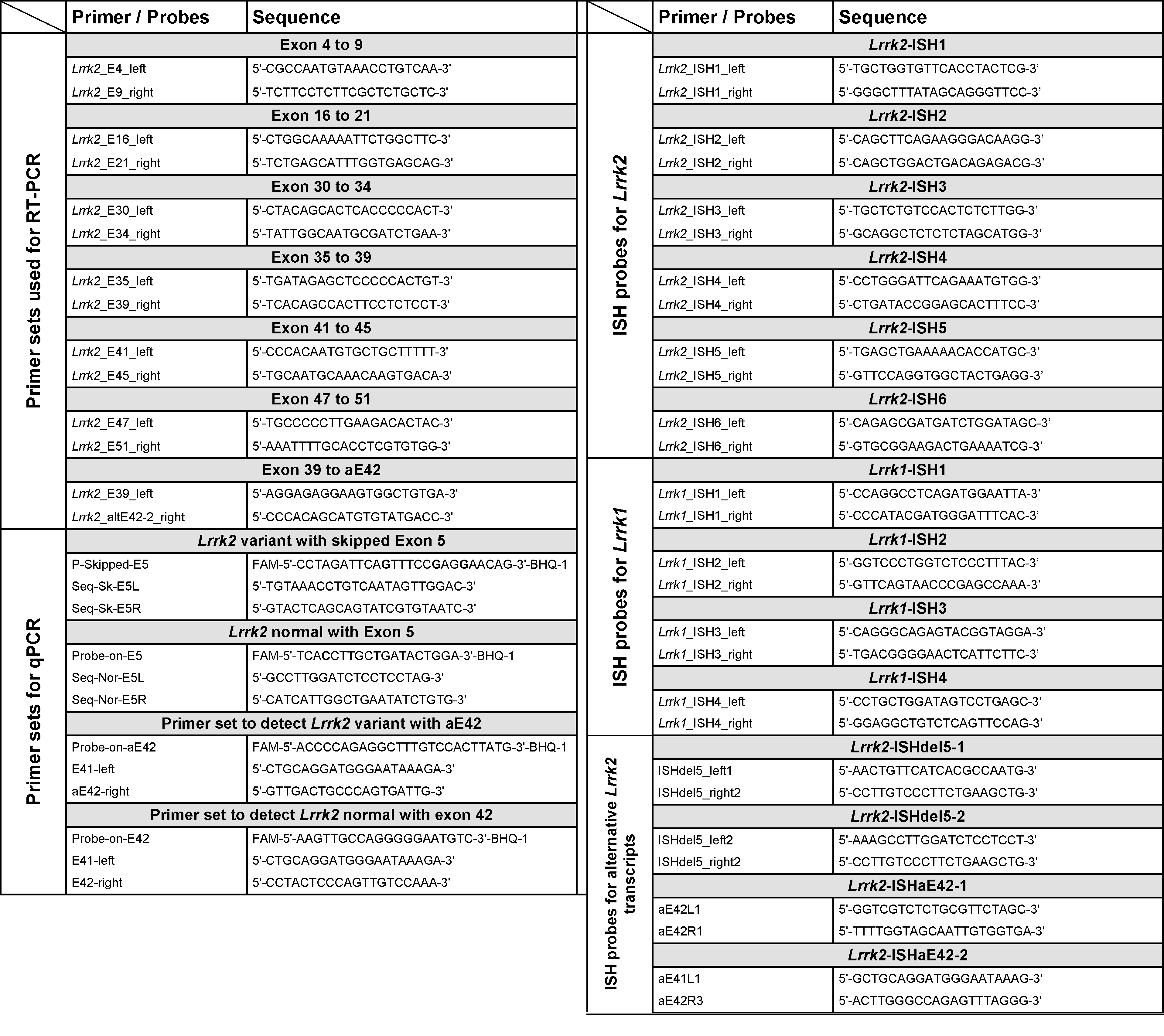

Supplement: Figure S9 — Sequence of primers and probes used in this study. Probes for qPCR were all 5′-labeled with 6-carboxyfluorescin (FAM) and 3′-labeled with Black Hole Quencher (BHQ-1). LNA-bases are indicated by bold letters. (TIF) [file pone.0063778.s009.tif]
